# Inflammatory and Microbiota-Related Regulation of the Intestinal Epithelial Barrier

**DOI:** 10.3389/fnut.2021.718356

**Published:** 2021-09-13

**Authors:** Giovanni Barbara, Maria Raffaella Barbaro, Daniele Fuschi, Marta Palombo, Francesca Falangone, Cesare Cremon, Giovanni Marasco, Vincenzo Stanghellini

**Affiliations:** ^1^IRCCS Azienda Ospedaliero-Universitaria di Bologna, Bologna, Italy; ^2^Department of Medical and Surgical Sciences, University of Bologna, Bologna, Italy; ^3^Medical-Surgical Department of Clinical Sciences and Translational Medicine, University Sapienza, Rome, Italy

**Keywords:** intestinal epithelial barrier, mucosal immune system, gut microbiota, IBS, IBD, celiac disease, non-celiac gluten sensitivity

## Abstract

The intestinal epithelial barrier (IEB) is one of the largest interfaces between the environment and the internal milieu of the body. It is essential to limit the passage of harmful antigens and microorganisms and, on the other side, to assure the absorption of nutrients and water. The maintenance of this delicate equilibrium is tightly regulated as it is essential for human homeostasis. Luminal solutes and ions can pass across the IEB *via* two main routes: the transcellular pathway or the paracellular pathway. Tight junctions (TJs) are a multi-protein complex responsible for the regulation of paracellular permeability. TJs control the passage of antigens through the IEB and have a key role in maintaining barrier integrity. Several factors, including cytokines, gut microbiota, and dietary components are known to regulate intestinal TJs. Gut microbiota participates in several human functions including the modulation of epithelial cells and immune system through the release of several metabolites, such as short-chain fatty acids (SCFAs). Mediators released by immune cells can induce epithelial cell damage and TJs dysfunction. The subsequent disruption of the IEB allows the passage of antigens into the mucosa leading to further inflammation. Growing evidence indicates that dysbiosis, immune activation, and IEB dysfunction have a role in several diseases, including irritable bowel syndrome (IBS), inflammatory bowel disease (IBD), and gluten-related conditions. Here we summarize the interplay between the IEB and gut microbiota and mucosal immune system and their involvement in IBS, IBD, and gluten-related disorders.

## Introduction

The gastrointestinal (GI) tract, with a surface area of about 40 m^2^, is one of the largest interfaces between the environment and the internal milieu of the body (1, 2). The intestinal epithelial barrier (IEB) is a structure that receives and reacts to several stimuli in a dynamic way (3), The IEB is essential as the first line of defense, limiting the passage of harmful microorganisms and antigens, and at the same time, it has to assure the correct absorption of nutrients, water, and ions. This delicate equilibrium is reached through the interaction of several elements: the mucus, the outer layer, followed by a single cell layer of epithelial cells and, finally, the lamina propria where immune cells, such as dendritic cells, plasma cells, macrophages, lymphocytes, reside (1). The intestinal epithelium, furthermore, includes five cell lineages: absorptive enterocytes, the most abundant epithelial cells, the goblet cells producing mucus (4), the tuft cells producing IL-25 (5), the enteroendocrine cells producing hormones, and Paneth cells, which produce antimicrobial peptides or lectins (4). The dysregulation of the IEB is implicated in the pathogenesis of several intestinal [e.g., celiac disease, inflammatory bowel disease (IBD), irritable bowel syndrome (IBS), colon carcinoma], and extra-intestinal diseases (e.g., chronic liver disease, type 1 diabetes, obesity). The hypothesis is that, for all these disorders, the IEB dysfunction, and consequent increase of intestinal permeability (i.e., a functional feature of the IEB, measurable by analyzing flux rates across the intestinal wall) (6), facilitate the entrance of antigens and/or microorganisms into the lamina propria with subsequent activation of the immune system and the initiation and/or maintenance of inflammatory responses (7, 8).

In the present review, we will summarize current evidence on the interplay between the IEB and immune system and microbiota and their involvement in GI pathological conditions including IBS, IBD, and gluten-related disorders.

## The Intestinal Epithelial Barrier

### Mucus

The mucus layer covers the luminal surface of the GI tract and is the first line of defense against mechanical, chemical, and biological insults. It protects epithelial cells from bacteria, digestive enzymes, and dangerous substances coming from the outside including environmental pollutants, food antigens, toxins (9, 10). In addition, it provides lubrication for food passage and removes bacteria and debris by flowing them away in the intestinal stream. Over then acting as a physical barrier, it is also essential in the maintenance of intestinal homeostasis as small molecules, gases, ions, and water diffuse through it to reach the epithelium (11, 12). It is continuously secreted in the GI tract principally by goblet cells, but also from epithelial cells and glands (12). Interestingly, the density of goblet cells increases distally within the GI tract and reaches the peak in the colon, likely in parallel with the increase of microbiota (13, 14).

Mucus is made up of water (90–95%) (11, 15) proteins, lipids (1–2%), and electrolytes (16). Proteins, principally produced by goblet cells, include mucins, which lend the mucus its gel-like properties. Mucus comprises also antimicrobial peptides and immunoglobulin-A (IgA), giving the mucus a pivotal role in innate defense (17–19). IgA are the most abundant isotype in humans and are secreted in the lumen where they are essential to prevent infections and to assure homeostasis with gut microbiota (20). The mucins are responsible for the gel aspect of the mucus and are characterized by mucin domains, rich in serine, threonine, and proline amino acids, that are abundantly O-glycosylated (21, 22). This post-translational modification gives these proteins the property to be soluble in water and to form a gel. In addition, the carbohydrates hide the protein core, preserving it from degradation. The glycan residues can bind the lectin-like proteins of immune cells, giving the mucus an active immunological role. In addition, mucin 2 (MUC2), the principal gel-forming mucin in the intestine, can influence dendritic cells and epithelial ones (23), contributing to oral tolerance [i.e., the unresponsiveness of the immune system induced by oral administrated innocuous antigens, including that derived from food (24) and gut homeostasis (25)]. Although mucus is present throughout the intestine, it has peculiar properties in the different tracts (26). In the small intestine, there is a single layer of mucus that is more permeable than elsewhere to contribute to nutrients' uptake (27). In addition, in this tract, mucus is discontinuous to support the release of digestive enzymes (27–30), it is not tightly attached to the epithelial cells, and it is mixed with antibacterial substances (31–33) which contribute to avoiding the contact between bacteria and epithelium (29, 34–36). In the large intestine, the mucus is divided into two layers: the inner and the outer ones. The inner layer is organized in lamellar layers made up of MUC2 multimers anchored to the epithelium and not accessible to bacteria (22, 27). In humans, at a distance of 200 μm from the epithelium, the action of proteases changes the structure of the inner mucus, producing the outer one. The outer layer (i.e., non-attached to the epithelium) is less dense than the inner mucus, has larger pores, and is the place where commensal bacteria live (37–39).

Some years ago, Kamphuis et al., proposed a new model of mucus. They characterized the colonic mucus barrier in rodents by using histological and FISH techniques. They demonstrated that in the distal colon, mucus covers the feces instead of epithelial cells confining the microbiota to the feces. On the other side, this organization is lost in the proximal colon, suggesting that mucus organization depends on the presence of colonic content (40).

The high polysaccharide content of the mucus represents a source of energy for bacteria, although only a subset of gut microbiota can hydrolyze mucus carbohydrates in physiological conditions (41, 42). Interestingly, in the case of a diet poor in fibers, there is a change in the microbiota composition, with an increase in the abundance of mucus-degrading bacteria that are capable to use glycosylated residues as a source of energy (43). Gut microbiota and its metabolites have a key role in the formation and correct folding of the mucus. As a matter of fact, germ-free mice showed a similar organization of the mucus layer to conventionally raised (Convr) mice, but the inner mucus was more penetrable, and small intestinal mucus was tightly attached to the epithelium. Following the colonization of germ-free mice intestine with Convr microbiota, colonic inner mucus became impermeable, and small intestinal mucus acquired the typical characteristic of being easily detachable (44). The mechanisms through which microbiota can influence mucus production and properties are still a matter of study. One possible way is that gut microbiota can influence mucin glycosylation (21) and the expression of glycosyltransferase. On the other hand, the gut-microbiota composition is influenced by mucus glycosylation pattern, which differs among species, establishing an equilibrium that ensures nutrients to microbiota and avoids mucus degradation (45). The role of mucus as a barrier is crucial for the maintenance of health. Reduction or abnormalities in the mucus production or in the glycosylation of the mucins are associated with gut inflammation and ulcerative colitis (46, 47) as well as in the colonization of the inner mucus by bacteria in both patients with ulcerative colitis and in the murine model of colitis (48). Qualitative and quantitative changes in the mucus layer in response to inflammation, have been demonstrated also in Crohn's disease (49–52) and colorectal cancer (53, 54). Finally, mucus is the first barrier for pathogens to enter the intestinal mucosa and start an infectious (55). Lipopolysaccharide (LPS) expressed on the outer membrane of Gram-negative bacteria, lipoic acid on the membrane of Gram-positive bacteria and flagellin, can activate MUC2 expression by the activation of toll-like receptors (TLRs) as reported in animal studies and in studies using cell lines, including goblet cell line and HT-29 (56–61). In conclusion, on the basis of the above evidence, the integrity of the mucus and the correct balance between mucus production by goblet cells and mucus degradation by intestinal microbiota, are essential for the maintenance of a health state.

### Tight Junctions

Beneath the mucus layer, there is a columnar monolayer of intestinal epithelial cells that represents an additional line of interface, between the outside environment and intestinal mucosa.

Alterations of the IEB allow the excessive passage of food and microbiota antigens which elicit immune activation involved in the pathogenesis of the intestinal and systemic disease (e.g., diabetes, obesity, non-alcoholic liver diseases). The functioning of the IEB depends on the presence of a series of intercellular junctions composed by the apical junctional complex (AJC), including tight junctions (TJs) and adherens junctions (AJs), and desmosomes. Under physiological conditions, only water and solutes like electrolytes can cross the epithelium through the paracellular way. TJs, the principal regulators of paracellular permeability (62), are a network of proteins located at the apex of the lateral membrane of epithelial cells, including claudins (63), tight junction-associated marvel proteins (TAMPs) such as occludin (64), junctional adhesion molecule-A (JAM-A) (65), and intracellular scaffold proteins, such as zonula occludens (ZO), and tricellulin (66).

Claudins are transmembrane proteins that form two extracellular loops interacting with their counterparts from the neighboring cell (67, 68).

Claudins are a group of proteins with molecular weights ranging from 21 to 34 kDa (68–70). Intestinal claudins exist in two functionally different classes: sealing claudins, responsible for the tightness, and pore-forming claudins (71). Sealing tight junction proteins include claudins-1, -3, -4, -5, -7, -8, -11, -14, -18, and -19 (68) and form a selective barrier to macromolecules and ions, whereas claudins-2, -10a/-10b, -15, -16, and -17 form selective pores to ions and water (72). Each pore-forming claudin has different specificity for cations or anions as well as selectivity for ionic size. The characteristics of each claudin are shown in Table 1.

**Table 1:** Characteristics of claudins and their changes in intestinal diseases.

**Claudin**	**Size (kDa) (68)**	**Localization**	**Functions**	**Ions permeability**	**Interactions (68)**	**Role in disease**
1	22.8	Ubiquitary (63)	Barrier forming	↓ Cations	It plays a general role in preventing the loss of water and macromolecules	↓ In UC (73–75), food allergy (76), liver cirrhosis (77), and IBS-D (78)
2	24.4	Intestinal crypts (79), Proximal renal tubule (80), choroid plexus (81), Human ovarian surface epithelium (82)	Pore forming	↑ Cations	It is involved in the regulation of Na^+^, Cl^−^, Ca^2+^ and water	↑ UC (83), celiac disease (84), IBS-D (85) ↑↓ Crohn's disease (86)
3	23.3	Human gallbladder (87) Brain capillary endothelium (88) Tighter segments of nephron (89) Liver/intestinal epithelial cells (79)	Barrier forming	↓ Cations	It is involved in the reduction of paracellular permeability of large molecules and in the formation of the blood-brain barrier	↓ Crohn's disease, acute colitis (90) ↑ Celiac disease (84)
4	22.1	Kidney and lung (68) Human gallbladder (87) Stomach, small intestine and colon (91)	Barrier forming	-	It can act as a Na^+^ barrier or, interacting with claudin-8, as an anion (Cl^−^) pore. In the colon strengthens tight junctions	↓ Acute colitis (73, 83) ↑ NCGS (92)
5	31.6	Endothelial tissue: endothelial cells (93) and brain capillary (94) Epithelial tissue: Human ovarian surface epithelium (82) Human colon epithelium (95)	Barrier forming	↓ Cations	It is involved in permeability of small molecules (≈800 Da)	↓ Acute colitis, Crohn's disease (86), Celiac disease (84, 96)
7	22.4	Epithelial tissue: Duodenum, jejunum, ileum, colon (97) Human palatine tonsillar epithelium (98) Nephron segments primarily at the basolateral membrane (99)	Barrier forming	↓ Anions	It plays a role in the regulation of Na^+^, Cl^−^, K^+^ and water	↓ UC (83), Celiac disease (84, 96)
8	24.8	Epithelial tissue: Duodenum, jejunum, ileum, colon (100) Distal nephron (99)	Barrier forming	↓ Cations	It can act as a Na^+^ barrier or Cl^−^ pore, depending on the cell type	↓ Crohn's disease (86)
12	27.1	Brain endothelial cells (94) Duodenum, jejunum, ileum, colon (97)	-	↑ Cations	It increases permeability to Ca^2+^	↑↓ Crohn's disease (101)
15	24.4	Kidney endothelial cells (89) Intestine (duodenum, jejunum, ileum, colon) (97)	Pore forming	↑ Cations	It can act as a Na^+^ channel or Cl^−^ barrier, depending on the cell type; it is involved in paracellular K^+^ absorption and Na^+^ secretion	↑ Celiac disease (84, 96)
18-2	27.7	Stomach (102) and bone cells (103)	Barrier forming	↓ Cations	It acts as selective barrier against Na^+^ and H^+^, protecting the epithelium from low pH	↓ Gastric cancer (104)

Occludin is a transmembrane protein with a molecular weight of around 65 kDa. It presents a long C-terminal cytoplasmic domain, two loops, and a short N-terminal cytoplasmic portion (64). The carboxy-terminal of occludin contains the binding site for zonula occludens (ZO)-1. Occludin is a phosphorylated protein and it has been reported that its phosphorylation correlates with the localization at the TJs (105). ZO-1 (~220 kDa), ZO-2 (~160 kDa), and ZO-3 (~130 kDa) are scaffold proteins, localized to the cytoplasm (106). ZO-1, -2, and -3 have three PDZ domains and a guanylate kinase-like domain (GUK) (107). PDZ1 binds the C-terminus of claudins (106) while the GUK domain interacts with occludin (107). C-terminal regions of ZOs interact with actin and serve as scaffolds linking TJ strands with the cytoskeleton (108–110). The association of the cytoskeleton with the TJ structure is crucial for the regulation and maintenance of TJs function (111). Junctional adhesion molecules (JAM, ~40 kDa) are transmembrane molecules localized to apical cell-cell contacts and associated with TJs. They are members of the immunoglobulin superfamily and include 3 transmembrane proteins JAM-A, -B, and -C (also called JAM-1,−2, and−3) characterized by a short N-terminal portion, two extracellular immunoglobulin-like loops, a transmembrane portion, a short cytoplasm fragment containing phosphorylation sites and a C-terminal PDZ domain involved in cell-cell adhesion (67, 112). The latter domain is involved in the binding of ZO-1 and is fundamental for protein-protein interactions (112–114). Among the JAM proteins, JAM-A is the best characterized in the regulation of TJ barrier function. It's expressed in intestinal epithelial cells and has also been implicated in cell proliferation and migration (115–118). The characteristics of ZO and JAM proteins are shown in Table 2.

**Table 2:** Characteristics of zonula occludens (ZO) proteins and junctional adhesion molecules (JAM) and their changes in intestinal diseases.

**Tight junction protein**	**Size (kDa)**	**Interactions**	**Role in disease**
ZO-1	~220 kDa	Claudin, JAM-A, occludin, F-actin, ZO-2,-3, cingulin	↓ Crohn's disease (119) IBS-D (120), IBS-A (121) Celiac disease (122)
ZO-2	~160 kDa	Claudin, occludin, F-actin, ZO-1,-3, cingulin	↓ IBS-D (120)
ZO-3	~130 kDa	Claudin, occludin, ZO-1, cingulin	↓ IBS-D (120)
JAM-A	~40 kDa	Occludin, JAM-A, ZO-1, cingulin	↓ In IBD (75, 83, 123) IBS-D and IBS-A (124)
JAM-B	~40 kDa	JAM-C, ZO-1	-
JAM-C	~40 kDa	JAM-B, JAM-C, ZO-1	-

Cingulin is a cytoplasmic protein with a molecular weight of 140–160 kDa. It has a structural role, binding ZOs and cytoskeletal proteins (125), and a signaling one, being involved in the control of epithelial proliferation (114, 126). TJs are linked to microtubules and microfilaments of the cytoskeleton. An important role in the regulation of TJ opening/closing is attributed to the microfilaments of actin and myosin (127, 128). Myosin is mainly involved in the regulation of TJ assembly and tone (1, 129), while actin binds to the cytoplasmic scaffold proteins (130). In addition, actin polymerizing proteins and regulators of actin, are central in TJ changes (114).

The composition and stability of the IEB depend not only on TJs but also on their interaction with AJs and desmosomes (131, 132). AJs establish cell-cell contacts, which are essential for TJs maturation and maintenance. E-cadherin is the main transmembrane protein involved in AJ assembly (133). Its intracellular domain is associated with p120, b-catenin, and a-catenin, forming a complex that is bound to actin filaments (134). Moreover, regulating the organization of the underlying actin cytoskeleton, it establishes a hub for cell signaling and regulation of gene transcription (135).

Desmosomes provide mechanical strength to the intercellular cell-cell contact in the epithelium. Their composition includes two subtypes of transmembrane cadherins, desmogleins, and desmocollins. These proteins through the binding with plakophilin and desmoplakin (136) can bind intermediate filaments providing junction stability to mechanical stress due to peristaltic movement of the gut (137).

Zonulin, the pre-haptoglobin 2, is an endogenous modulator of intestinal permeability.

The binding of gliadin to the chemokine receptor C-X-C Motif Chemokine Receptor 3 (CXCR3) on epithelial cells, likely induces the release of zonulin through a MyD88-dependent pathway. Zonulin disassembles tight junctions, through the transactivation of EGF receptor *via* proteinase-activated receptor 2 (PAR-2) activation (138, 139). Interestingly, zonulin serum levels are altered in conditions characterized by IEB alterations, including celiac disease (CD), type 1 diabetes, and non-celiac gluten sensitivity (NCGS) (138, 140, 141). It is to note that zonulin belongs to a family of structurally and functionally related proteins named “zonulin family peptides” that can affect intestinal permeability. In addition, it cannot be excluded that different members of this family could be detected by commercially available enzyme-linked immunosorbent assay (ELISA) kit (142).

### Transcellular and Paracellular Pathways

**Figure 1:**
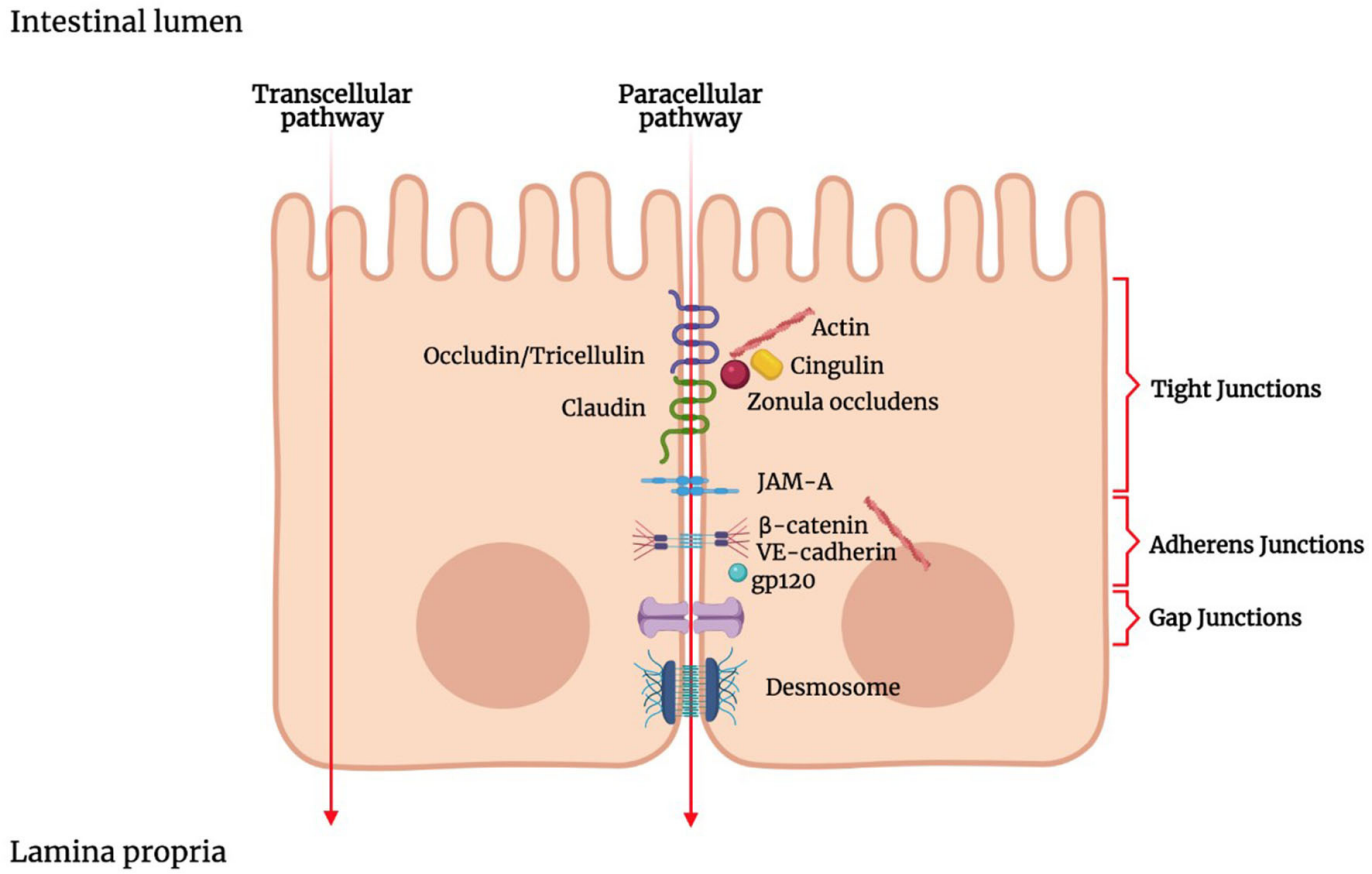
Schematic representation of the junctional complex in intestinal epithelium. Molecules can cross the intestinal epithelial monolayer through the intercellular space (paracellular route) or through the cells (transcellular route). The junctional complex involved in paracellular pathway includes tight junctions, adherens junctions, gap junctions and desmosomes. Tight junctions are composed of transmembrane proteins such as claudins, occludin, junctional adhesion molecule (JAM), and zonula occludens proteins (ZOs). ZOs, act as scaffold proteins, connecting claudins and occludin to cytoskeletal actin.

Luminal solutes and ions can pass across the IEB *via* two main pathways: the transcellular pathway (i.e., through the cells) or the paracellular pathway (i.e., between the cells) (Figure 1). The transcellular transport implicates the passage across the cell membrane, which generically occurs by passive transport, passive diffusion by efflux pumps, active transport, or endocytosis (143). Apolar compounds like soluble lipids can pass through the phospholipid bilayer by simple diffusion. Small hydrophilic compounds and nutrients mainly use channels and transporters located on cellular membranes to cross the cell membrane (144). Some channels belong to the group of the ligand-gated channels, which requires the binding of a ligand to the receptor region to modify their conformation and be opened or closed (145, 146). Carrier-mediated transport involves the binding of the target molecules to the receptor portion of the transporters, which in turn produces a conformational change in the carrier and allows the translocation of the compound (147).

Larger molecules, such as proteins and bacterial products, penetrate by the mechanism of receptor-mediated endocytosis, which implies the invagination of the plasma membrane and the subsequent formation of vesicles, as a consequence of proteins or peptides binding to specific cell surface receptors (148). The substances thus incorporated are transported through the cytoplasm by transcytosis. This transport is essential for antigen surveillance in the GI tract (149).

Endocytosis in epithelial cells occurs differently depending on the substance interacting with the epithelium. Immunoglobulins and viruses penetrate the epithelium *via* clathrin-mediated endocytosis. This is a highly specific receptor-mediated process in which molecules, after binding the receptor, are internalized *via* clathrin-coated vesicles (150). Transcytosis is a mechanism through which IgA can cross the IEB and arrives into the lumen. In particular, epithelial cells express the polymeric IgA-receptor (pIgA-R) that binds IgA. The extracellular portion of pIgA-R is cleaved at the apical side and released into the lumen with IgA (151), and the so generated secretory IgA (SIgA) complexes play multiple protective roles (152). Bacterial and food antigens delivery across the intestinal epithelium takes place throughout the transcellular pathway. At the level of Peyer's patches, SIgA can mediate antigens selective translocation, first binding them and then promoting their apical-to-basal transepithelial migration *via* M cells, dendritic cells, and T cells (20, 153–156). Whereas, the food allergens translocation throughout the intestinal epithelium, during the initial phase, involves transcytosis of IgE-allergen complexes mediated by the CD23 IgE receptor in epithelial cells (157, 158). Indeed, the IgE-allergen complexes binding to the CD23, located at the apical side of polarized epithelial cells, promote IgE-allergen-CD23 migration toward the basolateral surface of the cells with AP2-dependent endocytosis *via* clathrin-coated (159, 160).

Phagocytosis is another pathway that allows the uptake of antigens. In particular, enterocytes are able to carry out TLR-mediated phagocytosis to internalizing gram-negative bacteria (161). Moreover, phagocytosis is a route through which bacteria, viruses, and particles can enter enterocytes, after binding to different receptors (162, 163). Through the non-specific process of micropinocytosis, extracellular fluids are internalized, as well as dissolved molecules, viruses, and apoptotic cell fragments. Finally, there is the mechanism mediated by lipid rafts/caveolae, that seems to be involved in the internalization of some enterotoxins and viruses into enterocytes (151). This mechanism involves the invagination of cholesterol-rich areas of the plasma membrane that contain a coating protein, caveolin (164).

TJs are responsible for the sealing of the paracellular space between cells and, therefore, strictly limiting the transport of hydrophilic molecules (165–167). TJs have the so-called “gate and fence” function as they regulate the size and charge selectivity of the paracellular pathway. Indeed, TJs allow the paracellular transport of some medium-sized hydrophilic molecules, ions, or positively charged molecules but prevent the transport of proteins, such as antigenic macromolecules, lipids, and microbial-derived peptides (168).

## Gut Microbiota-Epithelial Barrier Interplay

The human gut harbors a community of about 10^14^ microorganisms resulting from thousands of years of co-evolution with the host, with an intricate and mutually beneficial relationship (169). Indeed, gut microbiota participates in digestive functions, shapes the host immune system (170, 171) modulates host metabolisms, and influences local and systemic processes, such as vitamin intake and nutrient transformation (172, 173). The intestinal microbial population also protects against pathogens, by competing with them for nutrient uptake and regulating host immunity (174–178). To avoid aberrant immune responses, the IEB separates microbes and immune cells, leading to the establishment of host-commensal mutualism (171). Despite this physical separation, gut microbiota can deeply modulate epithelial cells and the immune system (38) through the production of a series of metabolites. Microbial metabolites can be classified as metabolites generated by the host and then modified by the gut microbes into the lumen, such as secondary bile acids, dietary product-derived metabolites, such as compound K, or *de novo* synthesized metabolites such as short-chain fatty acids (SCFAs). SCFAs including butyrate, acetate, and propionate, profoundly influence aspects of GI physiology, such as contractility, visceral pain, epithelial proliferation, barrier function, host immunity, but also bacterial pathogenesis (179–181).

SCFAs can modulate the expression profile of epithelial cells, enhancing the production of proteins involved in the biosynthesis of mucin (182). Specifically, butyrate enhances MUC2 expression both activating the MUC2 promoter and enhancing histone acetylation by HDAC inhibition in cell cultures (183). Moreover, the intestinal epithelial cells express receptors activated by SCFAs. These are members of the G-protein coupled receptors (GPR) and include GPR41, GPR43, and GPR109a. The SCFAs binding to GPR41 and GPR43 stimulates colonic epithelium to releases chemokines and cytokines (184). The butyrate depending activation of GPR109a enhances IL-18 excretion in epithelial cells, protecting the colon against inflammation and carcinogenesis (185). Other microbial metabolites that significantly influence the maintenance of gut barrier integrity include indole derivatives, bile acid metabolites, conjugated fatty acids, polyamines, and polyphenolic derivatives. Their role in regulating gut barrier function was recently assessed in an extensive review (186).

TLRs are single-pass membrane-spanning receptors playing a key role in the innate immune system (187). They are expressed on the membranes of immune and non-immune cells, including epithelial cells, and recognize molecules that are broadly shared by microbes. In particular, TLR recognition of microbial fractions improves the IEB function, the secretion of mucus, and the production of antimicrobial peptides, promoting immune tolerance toward the gut microbiota. The role of epithelial TLRs in gut homeostasis and disease was recently reviewed (188).

Bacteria-epithelial cell interactions play an essential role in regulating epithelial permeability through the modulation of TJs expression and assembly (189). Animal studies clearly underlined the interplay between the intestinal microbiota and the IEB homeostasis. Germ-free mice had a higher colonic expression of claudin-1 and occludin as compared with conventional mice, with lower paracellular uptake of a standard probe, suggesting that commensal microbiota controls colonic TJs proteins and paracellular permeability (190). Interestingly, transplantation of fecal microbiota from healthy humans restores the IEB features of conventional mice within a week. In particular, colonization reestablishes physiological colonic paracellular permeability, maturation of colonic barrier structure, and reduces systemic microbial antigen exposure (190). Altogether these data suggest that gut microbiota is crucial in preserving the integrity of the IEB, thus preventing the systemic spreading of potentially harmful antigens.

Specific gut microbiota components may differently modulate the IEB permeability. For example, colonization of germ-free mice with *Bacteroides thetaiotaomicron* (191) or *Escherichia coli* Nissle 1917 (EcN) (192) led to up-regulation of genes encoding for proteins such as small proline-rich protein-2 (sprr2a) and ZO-1 involved in improving cellular adhesion. Interestingly, we have recently demonstrated that the increase in paracellular permeability elicited by supernatants obtained from patients with IBS could be ameliorated by EcN (193). Furthermore, EcN treatment abolished the correlations between increased permeability induced by IBS supernatants with abdominal pain and distension referred by IBS patients, paving the way to further explore clinical applicability of this probiotic in IBS human studies (193). In contrast, other bacteria had no impact on IEB permeability or could impair the IEB. For the latter, not only pathogens, such as enterohemorrhagic *Escherichia coli* (EHEC) O157:H7 (194), enterotoxigenic *Escherichia coli* (ETEC) K88 and *Salmonella typhimurium* SL1344 (195), but also non-pathogenic commensal bacteria, such as *Escherichia coli C25* (196), can be involved.

Growing evidence indicated that microbiota dysbiosis, occurring when the diversity, composition, and/or functions of the intestinal microbiome are disrupted, could contribute to the alteration of the IEB with implications in the development, progression, and symptom flare-up of several diseases. Among these, inflammatory conditions, such as IBD, and functional bowel disorders, such as IBS, are both characterized by a well-established microbiota dysbiosis associated with impairment of intestinal permeability. The interplay between the host, and in particular the semipermeable multi-layer ecosystem where intestinal epithelial cells exert a critical role, and the gut microbiota is constantly challenged by numerous factors, including genetics, age, environment, food, and immunological factors. A recent report of the Rome Foundation on the Intestinal Microenvironment and Functional Gastrointestinal Disorders (FGIDs) provided an excellent overview of the importance of the environment, including food, diet, microbiota, and its metabolic interactions, in the pathophysiology and symptom generation of patients with FGIDs (197). Patients with IBD display a loss of biodiversity (mostly Firmicutes) and stability, while an increase of Proteobacteria such as *Enterobacteriaceae, Bilophila*, and certain members of Bacteroidetes [for review, see (198)]. In addition, *Akkermansia muciniphila*, a mucolytic commensal, is generally reduced in the gut of these patients, with a consequent increase of the overall mucosal bacteria population (42). Furthermore, patients with IBD display a reduction in SCFA-producing bacteria such as *Faecalibacterium prausnitzii* (199) that is well-known to have anti-inflammatory properties through its ability to produce butyrate, allowing for T regulatory cell and T helper 17 regulation (200). Altogether, these changes may lead to a loss or reduction of key functions necessary for maintaining IEB integrity, potentially resulting in increased immune responses and the diffusion of pathogens into the intestinal tissues. In addition, bacterial translocation induces the production of inflammatory cytokines, which promote the disassembly of TJs, enhancing IEB permeability. However, if these changes are a cause or consequence of these diseases, this is virtually unknown at this time. Strategies to improve the relationship between host and intestinal microbiota to enhance epithelial mucosal permeability include the use of probiotics and dietary interventions, although there is still uncertainty on the potential benefits (6). In this context, we have recently demonstrated that *Lactobacillus paracasei* CNCM I-1572 modulates gut microbiota structure and function and reduces intestinal immune activation in patients with IBS. Interestingly, the most robust result was obtained for a marked reduction of interleukin 15 (IL-15) that affects the integrity of the IEB, suggesting that this probiotic may play an important role in the restoration of mucosal integrity (201).

## Gut Immune System-Epithelial Barrier Interplay

The integrity of the IEB depends on the delicate balance between differentiation and renewal of intestinal epithelial cells, the response to signals coming from the lumen including microbiota and their end products, and nutritional factors introduced with daily diet as well as signals coming from the mucosal immune system (202). Several factors, including cytokines, proteases, growth factors, gut microbiota, and dietary components are known to regulate intestinal TJs opening (6). Immune dysregulation in several disorders of the gastrointestinal tract such as IBD, celiac disease, colonic cancer, and IBS is often associated with impaired IEB integrity or dysfunction. Indeed, the IEB may represent the target of mediators released by inflammatory cells in the lamina propria. This elicits epithelial cell damage and TJ dysfunction, mucus structural and functional alterations, ultimately leading to increased intestinal permeability (6). Disruption of the IEB would then allow the passage of antigens, bacterial products in the mucosa leading to further inflammation hence creating a self-maintaining pathological inflammatory process (6). The involvement of the immune system in conditions affecting the gastrointestinal tract can be greatly different for magnitude as well as the type of immune cells. Conditions characterized by mucosal inflammation often show the involvement of both innate as well as adaptive immunity.

Intestinal epithelial cells and mononuclear phagocytes sense bacteria or their products mainly through pattern recognition receptors such as TLRs (166). Downstream of activation of innate responses is the production of cytokines, including the IL-1 family cytokines such as IL-1 and IL-18 which lead to pro-inflammatory effects in the context of intestinal inflammation. IL-1 can further promote Th17 cell differentiation and IFN-γ production from T cells (203) and its two isoforms (IL-1α and -β) seem to be involved in epithelial repair/regeneration (204). IL-1R1 is a receptor expressed on different cell types of the colon such as innate lymphoid cells (ILCs) and GREM+ mesenchymal cells (205). Cox et al. demonstrated that in a mice model of DSS-induced colitis, the binding of IL-1α/β to IL-1R1 in ILCs induces the production of IL-22, a cytokine involved in progenitor cell proliferation. Conversely, in a model of *C. rodentium* infection, the activation of IL-1R1 occurs both in ILCs and in GREM+ mesenchymal cells, inducing the production of R-spondin 3 (RSPO3), an intestinal stem cell self-renewal activator. The IL-1R1-dependent response thus appears to reveal a damage-specific reparative capacity, which opens the door to IL-1-based therapeutic approaches (206).

Other important cytokines in the orchestration of innate immunity-related intestinal inflammation, include IL-6, IL-33, TNF-α (202). The release of TNF-α and IFN-γ in the intestinal mucosa has been associated with IEB disruption in patients with IBD (207–210) as well as IBS (211). In line with this concept, incubation of intestinal epithelial cell monolayers with TNF-α and IFN-γ elicits profound changes of claudins, occludin, ZO-1, JAM-A, leading to increased epithelial permeability (212). During intestinal inflammation, TJs show strand breaks, and changes in TJ proteins composition and function as well as impaired structure and remodeling of apical junctions (213).

During inflammatory processes neutrophils, mast cells, and macrophages release proteases (214). Besides degrading the extracellular matrix and proteins, proteases act as signaling molecules *via* specific receptors (215). Proteases may deeply influence the integrity of the IEB either through excessive proteolysis determined by direct cleavage of intercellular junction proteins, or by a functional TJ opening elicited by the activation of protease-activated receptors (216). Mast cells are very proficient producers of proteases and mast cell activation has been proposed as underlying pathogenetic mechanisms in different gastrointestinal disorders, including IBS (217, 218).

## Clinical Implications

### Irritable Bowel Syndrome

**Figure 2:**
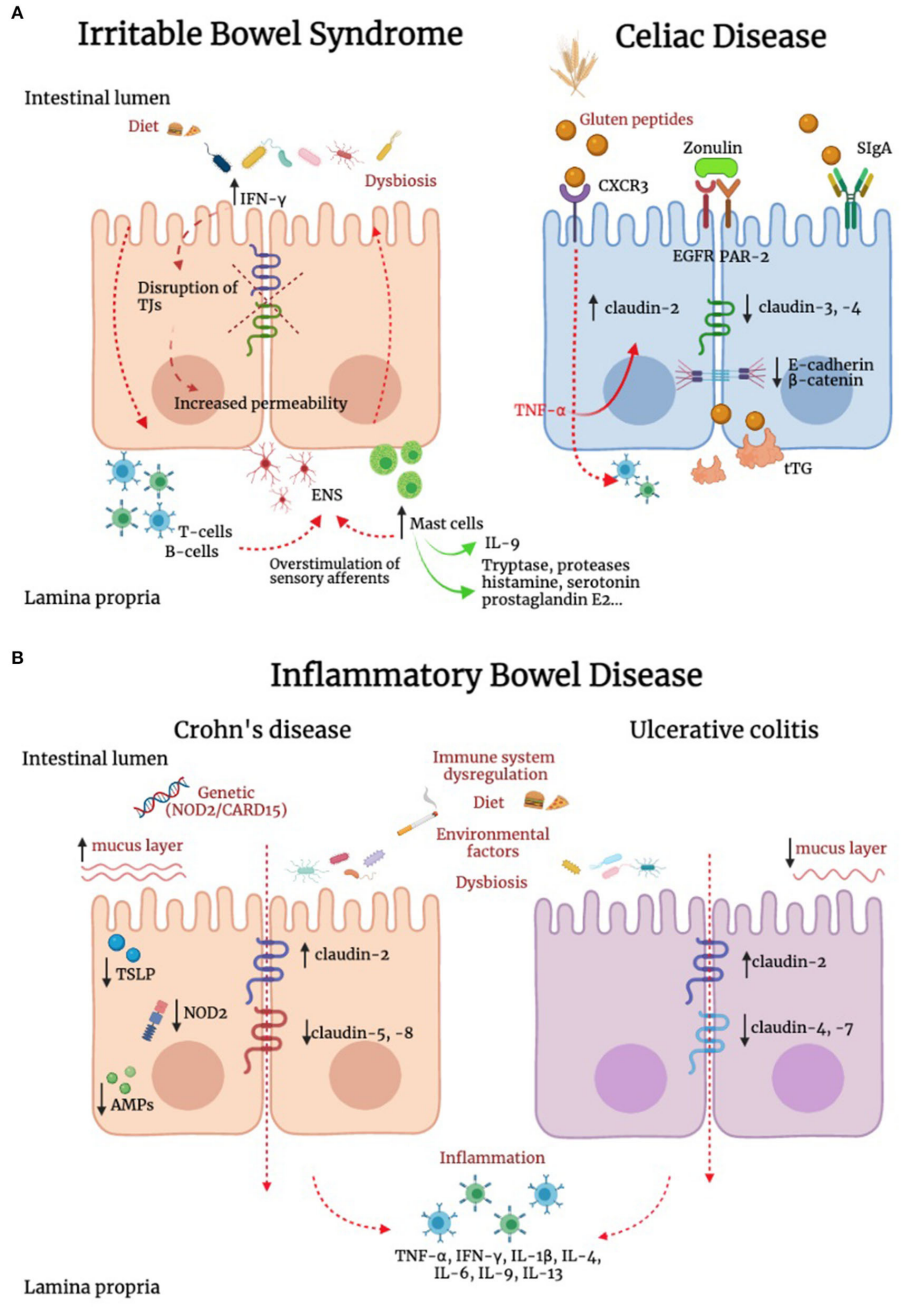
Alteration of IEB in irritable bowel syndrome, inflammatory bowel disease and celiac disease. **(A)** In irritable bowel syndrome (IBS), tryptase, histamine, and interferon-γ (IFN-γ) are increased and can contribute to TJ disruption. In addition, IL-9 is produced by innate lymphoid cells, T helper cells and mast cells. This latter cell population has a key role in IBS pathophysiology, and can modify TJ protein expression. In addition, immune mediators, including histamine, serotonin and proteases evoke sensory afferent over-stimulation and contribute to symptom generation. In celiac disease (CD), intestinal epithelial cells recognize gluten peptides through CXCR3, which increases IEB permeability through zonulin release and its transactivation of epidermal growth factor receptor (EGFR) and protease activated receptor 2 (PAR-2). Transcytosis of gluten peptides occurs after peptide recognition by secretory immunoglobulin A (SIgA). A reduction in the expression of E-cadherin and β-catenin was found in intestinal biopsies of CD patients. Active tissue transglutaminase2 (tTG2) deamidates gluten peptides, contributing to the development of a cell mediated pro-inflammatory immune response. **(B)** Genetics, environment, diet, immune system dysregulation and dysbiosis represent some of the complex mechanisms responsible for inflammatory bowel diseases (IBD). Inflammation down-regulates TJs proteins contributing to IEB alteration. In IBD patients has been reported an upregulation of the pore-forming claudin-2 and downregulation of occludin. Channel-forming claudins are up-regulated by cytokines including TNF-α and IL-13, leading to an increased permeability for ions and water. Occludin is down-regulated by inflammatory processes (e.g., TNF-α and IFN-γ), leading to increased paracellular permeability for macromolecules. Moreover, it has been shown a downregulation of claudin-5 and -8 in Crohn's disease and claudin-4 and -7 in Ulcerative Colitis (UC). In Crohn's disease, the stimulation of NOD2, a sensor of Gram-positive bacteria, induces the production of pro-inflammatory mediators, which concur to IEB dysfunction. Differently from UC, in Crohn's disease the mucus layer is thicker, suggesting an increase in MUC2 expression and goblet cells hyperplasia. In patients with Crohn's disease, intestinal epithelial cells (IECs) failed to produce thymic stromal lymphopoietin (TSLP), with consequent inability to control IL-12, produced by dendritic cells and involved in the development of Thelper 2 cells, resulting in alteration of intestinal homeostasis. Compared to UC, in which the antimicrobial peptides (AMPs) system seems to be adequately induced, Crohn's disease is characterized by lower levels of AMPs. Contrasting evidence are available on the role of smoking in UC and Crohn's disease.

Differently from IBD and celiac disease, IBS is not associated with overt mucosal inflammation, and evidence supporting the potential role of cytokines in these patients has been so far controversial (Figure 2) (219–221). However, we previously reported that IBS is associated with a marked increase in IFN-γ gene and IFN-γ protein expression in the colonic mucosa (211), a finding also supported by evidence of increased IFN-γ release in the lumen (222). As IFN-γ is known to increase paracellular permeability through disruption of TJs (223). This cytokine could well be involved in increased paracellular permeability described in patients with IBS (224). In addition to IFN-γ, other players could be of relevance in the dysregulation of TJs. IL-9 is abundantly produced by mast cells as well as innate lymphoid cells and T helper cells. Evidence suggests that IL-9 amplifies intestinal mastocytosis involved in several inflammatory diseases of the gastrointestinal tract (225). Recently, it has been shown that IL-9 modulates IEB function, modifying TJs proteins expression. In particular, IL-9 increases intestinal permeability through the upregulation of claudin-2 expression in an experimental model of ulcerative colitis (226). This concept is further supported by recent studies showing that in murine models of colitis, the TJ protein claudin-1 showed lower expression levels in IL-9 knock-out mice (227).

We have previously shown that the permeability of colonic biopsies was significantly higher in patients with IBS compared to healthy subjects (228). Tryptase, a key serine protease released by mast cells upon degranulation, was abundantly increased in mucosal biopsy supernatants of patients with IBS compared to healthy controls (229–231). Interestingly, the application of mucosal biopsy supernatants of IBS patients elicited a marked increase in paracellular permeability in Caco-2 epithelial cell monolayers (193). The importance of proteases in the pathogenesis of IEB dysfunction described in IBS is further supported by the evidence that transfer of fecal supernatants from patients with diarrhea-predominant IBS evoked increased mucosal permeability in mice and mucosal factors obtained from IBS evoked IEB dysfunction and TJ disruption in isolated epithelial monolayers (228). The effect of fecal supernatants on the epithelial barrier was absent in mice lacking PAR-2 (232). These results suggest that mucosal or luminal mediators impact negatively IEB by increasing epithelial permeability through a protease, PAR-2-dependent pathway and open new avenues for therapeutic intervention (232).

Contrary to the long-lasting belief that IBS is not associated with organic changes, there is now growing evidence supporting that structural, although subtle, changes may be found in the gastrointestinal tract of large subgroups of these patients (197). The observation that IBS can develop after an episode of acute infectious gastroenteritis further supports the organic as opposed to the functional nature of this condition (233). Many studies showed that the intestinal mucosa of IBS patients contains larger numbers of immune cells and evidence of a higher state of activation of the immune system (218). Interestingly, growing evidence supports the concept that IEB dysfunction may be key in the initiation and progression of immune activation and that this may eventually contribute to brain-gut axis dysfunction and symptom generation (228, 234). Accordingly, this evidence has generated a new dogma in the pathophysiology of IBS. More in detail, microenvironmental factors (e.g., food, microbiota, bile acids) would permeate in excess through a leaky IEB, allowing amplification of signaling from the lumen to deeper mucosal and muscle layers, including overstimulation of the mucosal immune system (197). These factors may determine abnormal signaling to neural circuits (intrinsic primary afferent nerves and extrinsic primary afferent nerves), which in turn may affect intestinal physiology and sensory perception (197).

Nonetheless, evidence indicates that there is a poor correlation between pathogenetic mechanisms and symptom generation in IBS. More likely, only the combination of peripheral (i.e., intestinal) factors, in conjunction with central nervous system mechanisms is necessary for the full expression of symptom perception. For this reason, IBS, like many other functional gastrointestinal disorders, is now better defined with the new term of Disorders of Gut-Brain Interaction (DGBI). On the same line, a single phenomenon is insufficient to explain the complexity of the protean symptom experience of IBS. One example in line with these concepts was our previous demonstration that the number of mucosal mast cells was increased in the colonic mucosa of patients with IBS (229). However, this phenomenon alone was not correlated to any symptom experienced by the patients. Nonetheless, when this parameter was associated with the presence of activated mast cells in close proximity with nerve endings in the mucosa of the intestine, we found a stringent correlation with abdominal pain (229). In addition, there is wide redundancy in pathogenetic mechanisms, and the different pathogenetic mechanisms influence each other and therefore should be always considered in context. For example, on one hand, changes in gut microbiota can promote IEB dysfunction which can lead to mucosal inflammation. On the other hand, inflammatory mediators can increase mucosal permeability and influence gut microbiota. This generates a vicious and integrated circle that cannot be separated into fully independent compartments.

With all the above considerations in mind, one of the most common observations in the studies evaluating the gut IEB and IBS relates to the correlation between increased intestinal permeability and abdominal pain (234). The link between epithelial permeability and pain likely reflects the abovementioned redundancy of the system involving the gut microbiota overstimulating the immune system through a leaky IEB, leading to the release of immune mediators, such as histamine, tryptase, serotonin, polyunsaturated fatty acids (12-hydroperoxyeicosatetraenoic acid, 15-hydroxyeicosatetraenoic acid, 5-hydroxyeicosatetraenoic acid, 5-oxoeicosatetraenoic acid, and leukotriene B4) (235, 236) known to evoke sensory afferent over-stimulation and pain (230).

Although some tantalizing findings have been reported, the final proof to demonstrate the key role of increased intestinal permeability in IBS has yet to be provided. There is a need for longitudinal studies that include assessment of the IEB function over time and its correlation with symptoms in well-characterized IBS populations. In addition, further studies assessing the role of IEB modulation in IBS are now needed. In this perspective, a recent study using a medical device containing xyloglucan, pea protein and tannins from grape seed extract, and xylo-oligosaccharides, acting together to protect and reinforce the IEB, effectively controlled diarrhea and alleviated clinical symptoms in patients with IBS-D (237).

### Gluten-Related Disorders

Gluten-related disorders comprise distinct clinical entities, including celiac disease (CD), wheat-associated allergy, and NCGS, characterized by distinct pathophysiological pathways, with altered permeability following gluten ingestion as a possible common shape.

CD is a chronic systemic disorder triggered by the abnormal response of human immunity to gluten ingestion in genetically pre-disposed individuals (238). The activation of the immune system against gluten peptides begins after their transfer to the lamina propria where they are deamidated by the tissue transglutaminase enzyme and subsequently bounded to human leucocyte antigens (HLAs) expressed by antigen-presenting cells (APCs), which activate the inflammatory cascade (Figure 2) (239, 240). However, the presence of gluten and genetic background does not fully explain CD pathogenesis. Indeed, in studies on twins, in one-fourth of cases, only one twin developed CD (241). Mounting evidence suggests that CD onset is favored under the influence of triggering environmental factors, such as viral infections and dysbiosis (242–247) which activate innate immunity leading to IEB disruption (248). Several studies suggest that the *primum movens* for CD onset depends on an increased intestinal permeability, which leads to gluten passage from the lumen to the mucosal layer and innate cytotoxic immune activation on epithelial cells further enhancing gluten migration (249). Although the IEB has been extensively investigated in the context of CD pathogenesis, to date is still unclear whether the IEB impairment is a cause or consequence of CD. On the other hand, in the last decades a novel clinically entity has been defined in patients without CD complaining extraintestinal and gastrointestinal symptoms referring to a clear benefit by avoiding gluten from their diet and/or symptom worsening upon gluten reintroduction, namely NCGS (141). The pathophysiology of NCGS is incompletely known, although some evidence suggests a role for permeability alterations, microbiota changes, and immune activation (141). The Salerno criteria are the gold standard to diagnose NCGS. These criteria imply a double-blind placebo-controlled gluten challenge (250), which is cumbersome and unfeasible to perform in clinical practice. In addition, there are no available biomarkers for NCGS. For all these reasons, diagnosis is currently based on self-reported symptoms by patients. There is a great overlap in symptoms between NCGS and IBS which makes a challenge the differential diagnosis between these two conditions. We have recently reported that zonulin serum levels are significantly increased in NCGS compared to IBS. In addition, the combination of zonulin levels, gender, and abdominal symptoms, can differentiate NCGS from IBS with a diagnostic accuracy of 89% (141).

Studies carried out using epithelial cell lines and animal models showed that gliadin was able to induce apoptosis of intestinal epithelial cells through a direct toxic effect (251), including enterocyte atrophy, villi shortening, decreased epithelial disaccharidase activity, increased expression of HLA molecules, and intraepithelial inflammatory activation. Among other effects, gliadin fraction p31-43 is able to mimic epidermal growth factors in human intestinal epithelial cell lines, finally leading to barrier defects (252). This peptide is usually detoxified during the transport in healthy subjects, whereas in active CD patients most of these peptides are transported intact in the serosal compartment; this process may be explained by the binding between gluten peptides with anti-gliadin secretory IgA finally forming large complexes in the intestinal lumen which in turn bind the CD71 receptor, namely the endocytic receptor for transferrin, thus activating the transport across enterocytes escaping lysosomal degradation (156). It is unclear whether this process is involved in CD development since it has been postulated that the entrance of IgA-gliadin complexes may break local immune tolerance (253). However, it is more likely that this process may perpetuate the immune reaction against gluten once the disease started. Besides, the effect of gliadin on the enterocyte actin cytoskeleton was studied on rat intestinal epithelial (IEC-6) cell cultures, finding that it was able to reversible stimulate tyrosine phosphorylation of actin filaments resulting in filaments polymerization, cytoskeleton reorganization, and tight junction opening (254). Other authors suggested that gliadin was able to bind the mono-sialic ganglioside 1 (GM1) or the receptors for the proinflammatory chemokine 3 (CXCR3) in mouse models, leading to the release of zonulin which in turn decreases electrical resistance of epithelial layers, finally resulting in increased epithelial permeability (139, 255). Indeed, zonulin release can induce cytoskeleton reorganization and zonula occludens-1 and occludin downregulation (139, 256). More interestingly, the receptors for the proinflammatory chemokine CXCR3 were also activated by the overexpression of its chemokine ligand 10 (CXCL10), which in turn is upregulated *in vitro* by viral infections (256).

Macroscopic alterations in intestinal permeability have been studied since the 60s (257) using tests able to measure the absorption of two inert probes of different dimensions by the IEB (257). The first probe was mannitol, which was taught to get through the IEB freely; the second was lactulose, which was able to get through the IEB only when its integrity was lost. An increased ratio between urinary lactulose/mannitol concentrations 6 and 12 h after oral administration indicated increased intestinal permeability, which has been extensively reported in CD patients (258–260). These alterations were also correlated to villus atrophy and intraepithelial lymphocyte count. Indeed, a further study showed that permeability increase was dependent on the release of IFN-γ by intraepithelial lymphocytes (207).

Structural changes consisting of dilatation, destruction, and reduced number of TJ strands were found in CD patients from ancillary (261) to more recent studies using transmission electron microscopy of duodenal biopsies (262). In CD has been widely reported an increased expression of pore-forming proteins such as occludin and claudin-2, together with a decrease of pore-sealing proteins such as claudin-3 and -4 and ZO-1 (84, 262–264). Claudin-2 expression is also induced by TNF-α, which in turn is upregulated in CD (86) and responsible for increased transcellular permeability in CD. These alterations are simultaneous to the loss of the penta-laminar ultrastructure and dilatation of TJs found in CD (262). As matter of fact, the overexpression of pore-forming and under-expression of pore-sealing TJ proteins is responsible for the dilatation of TJs (262).

CD patients have been found to have also alterations in adherence junction protein expression. A reduction in the expression of E-cadherin and β-catenin was found using transcription analysis in both intestinal biopsies of CD patients and Caco-2 cells (265). Moreover, an impaired interaction between ZO-1, mostly not phosphorylated in CD patients, and β-catenin has been reported, finally, leading to an absent connection between β-catenin and occludin in CD patients (263). On the other hand, β-catenin has been reported to be highly phosphorylated in CD, thus not binding E-cadherin which in turn is free to bind intra-epithelial lymphocytes responsible for inflammatory activation (263). Together with barrier impairment, epithelial polarization may also affect permeability since it regulates the function of proteins partition defective-3 (PARD-3) and protein phosphatase-2 (PP-1) which are involved in tight junctions formation (266).

Although these alterations are not specific for CD and may merely depend on mucosal inflammation, tight junctions' abnormalities have been found even in asymptomatic and first-degree relatives of CD patients, thus underlying a possible genetic background for permeability defects in CD (267). Supporting this hypothesis, polymorphisms in tight junctions encoding genes, such as PAR-3, membrane-associated guanylate kinase WW, and PDZ domain containing 2 (MAGI-2) and myosin IXb (MYO9B) have been found in CD patients (268–270). A subsequent study did not confirm a direct causal relationship between genetic polymorphisms and increased intestinal permeability (271). However, more recent studies evaluating CD loci showed a possible role of other genes involved in barrier functions and cell-cell adhesion (272–274).

To date, the only effective treatment for CD is a gluten-free diet (GFD). GFD is able to partially restore TJs abnormalities found in CD since TJs numbers remained low in crypts (261). Other studies found a normalization of ZO-1 (264) expression after gluten removal *in vitro* and of claudin expression (262) after 6 months of GFD. In parallel, *in vitro* models showed that β-catenin and E-cadherin alterations were reversible after gluten removal (265, 275). These microscopic findings were further confirmed by the recovery of a normal lactulose/mannitol ratio after histological mucosal healing due to long-term GFD (276). This evidence additionally confirmed that most intestinal permeability alterations were reversible, thus excluding the previous hypothesis of a causal role for genetic background. Additional or alternative therapeutic options to GFD focused on the reduction of intestinal permeability have been proposed for CD patients. Gluten detoxification through proteolysis or gliadin sequestrants has been proposed for overcoming the gliadin-related toxic effect across the epithelium (277, 278). A randomized controlled trial carried out in 2007 aimed to improve intestinal permeability using larazotide acetate (AT-1001), which is an inhibitor of paracellular permeability derived from a protein secreted by *Vibrio cholerae* and analogs of human zonulin (279). The authors concluded that larazotide acetate reduced IEB permeability assessed by lactulose/mannitol permeability test, proinflammatory cytokine production, and gastrointestinal symptoms in CD after gluten exposure (279). Two further phase II trials (280, 281) with larazotide acetate failed in finding an improvement in intestinal permeability, whereas the last trial published (282) underlined possible usefulness in refractory CD, defined as malabsorption and continued villous atrophy on duodenal biopsies despite strict avoidance of gluten for a minimum of 12 months (283). Another promising approach for reducing intestinal permeability is the use of probiotics, which however deserve further studies (244, 284).

### Inflammatory Bowel Diseases

Dysfunctional IEB has been implicated as a pathogenic factor in IBD in the last 30 years (285, 286). IBD includes a spectrum of disorders such as Crohn's disease and ulcerative colitis (UC), representing chronic remittent or progressive conditions determined by non-specific inflammation and intestinal tissue damage. It has been characterized by exaggerated and inappropriate mucosal immune responses that can involve the entire mucosal wall, as in Crohn's disease, or be confined to the submucosa, as in UC (287). The pathogenesis of IBD is complex and multifactorial and it is not fully understood, involving a complex dysregulated interaction between different factors (Figure 2). Particularly genetic pre-disposition, gut microbiota, and innate and adaptive immune responses represent fundamental elements (288). Some authors suggest that the alteration of the balance between these three components would be responsible for the triggering of the inflammatory environment needed to induce IBD (289). Otherwise many researchers reported that many other factors are involved in the pathogenesis of IBD such as dysfunction of intercellular transport mechanisms (290, 291), associated with factors responsible for the exacerbation in IBD (i.e., cigarette smoking, diet, stress, microbial dysbiosis, and food additives) (292–297). In fact, urbanization of societies is associated with changes in diet, antibiotic use, hygiene status, microbial exposures, and pollution, which have been implicated as potential environmental risk factors for IBD (298). Although these factors have been explored, the data available are still inconclusive (299). It is still a matter of debate the role of smoking in IBD. Evidence reported in literature shows smoking is associated with opposing risks in Crohn's disease and UC (300–302).

A meta-analysis focused on the effects of smoking behaviors and IBD has demonstrated that different races may have varying degrees of susceptibility to IBD (300). Smoking may play differing roles in the development of Crohn's disease compared with UC. Studies that involve Israeli populations, for example, have consistently failed to demonstrate a positive association between smoking and Crohn's disease, yet these same studies have shown a protective relationship between smoking and the development of UC (300). Conversely, a recent paper showed current smokers compared to never-smokers had an ~2-fold risk of UC and Crohn's disease (303).

Another study analyzed systemic concentrations of key chemokines and cytokines in IBD patients with a different range of disease activity compared to levels found in healthy donors (304). The result shows that there was a significant increase of chemokines including macrophage migration factor (MIF), CCL25, CCL23, CXCL5, CXCL13, CXCL10, CXCL11, MCP1, and CCL21 in IBD patients as compared to normal healthy donors. Further, has been reported an increase in the inflammatory cytokines IL-16, IFN-γ, IL-1β, and TNF-α in IBD patients when compared to healthy donors (*P* < 0.05). These data clearly indicate an increase in circulating levels of specific chemokines and cytokines that are known to modulate systemic levels through immune cells, results in affecting local intestinal inflammation and tissue damage in IBD patients (304). More recently it has been studied the role of antimicrobial peptides (AMPs) in IBD patients. Between this group of molecules, the most important ones are produced in the gut epithelium and are α-defensins HD5 and HD6, produced by the small-intestinal Paneth cells, and ß-defensins (constitutive HBD1 and inducible HBD2 and HBD3), mostly in the gastric and colonic epithelium (305). The changes in the microbiome composition and the bacterial contamination of the mucosa as well as the inner layer of the mucus may well be mediated by defects in this chemical defense (306). Compared to ulcerative colitis, in which the AMP system seems to be adequately induced, colonic Crohn's disease is characterized by low HBD1, regulated by peroxisome proliferator-activated receptor gamma (PPARγ), and a compromised induction of HBD2 and HBD3 (307, 308). Considering the important role played by the intestinal epithelium, which establishes a tightly regulated barrier, its integrity defects are frequently reported during intestinal inflammation (309). Microscopic analysis of intestinal tissue from IBD patients reveals a decrease in goblet cells (50), reduction thickness of mucus layer, and defective defensin production (310), with an altered composition of some components such as mucins and phosphatidylcholine (311). The alteration of the mucus protective barrier plays a key role in the onset of IBD (312). A recent case-control study reported that mucus abnormalities contribute to UC pathogenesis, demonstrating how core mucus structural components were reduced in active UC (313). These alterations were associated with attenuation of the goblet cell secretory response to microbial challenge and occurred independently of local inflammation. Another important mechanism involved in the alteration of the mucus protective barrier in IBD is the invasion by bacteria directly in contact with the epithelium and their penetration into the crypts and epithelial cells (314). This mechanism has been demonstrated in mice lacking MUC2 mucin (27). In these animal models, bacterial invasion induces the response of the colonic immune system resulting in inflammation, spontaneous colitis, diarrhea, rectal and colon prolapse, rectal bleeding, and an increased risk of colon cancer development. Differently from UC, in Crohn's disease, the mucus layer is thicker, suggesting an increase in MUC2 expression and goblet cells hyperplasia. Nevertheless, the structure of MUC2 is altered due to a reduction in the oligosaccharide chain length by 50%, leading to a loss of mucus viscoelastic properties and consequently a loss of protective function (315). Furthermore, indirect data suggest that barrier defect might precede the onset of disease (316) and up to 40% of first-degree relatives of patients with Crohn's disease demonstrate an altered small intestinal permeability (317). Moreover, it was shown that patients that had increased intestinal permeability were at greater risk of successive disease relapse (318, 319).

The mechanisms involved in the proper functioning of the IEB include the transport of molecules across the intestinal mucosa through two distinct mechanisms: paracellular diffusion through TJs between adjacent intestinal epithelial cells and transcellular transport involving the transcytosis of materials, eventually mediated by membrane receptors. In IBD patients has been observed increased paracellular permeability with abnormal TJ structure and a down-regulation and redistribution of many TJ proteins or other adherents junctions (320, 321). Both paracellular hyperpermeability [demonstrated by abnormal TJ expression and upregulation of myosin light chain kinase (MLCK) activity (86, 322–324)] and transcellular hyperpermeability [represented by bacterial internalization to epithelia (325)] were documented in mucosal biopsies of patients with Crohn's disease and UC. Disruptions of TJ proteins could lead to an alteration of the IEB, allowing entry of luminal bacteria. In fact in IBD has been reported an upregulation of the pore-forming claudin-2 and down-regulation of occludin, particularly claudin-5 and -8 in Crohn's disease and claudin-4 and -7 in UC (83, 86, 326). Increased expression of claudin-2 determines an increased number of pores responsible for the paracellular movement of small molecules, characteristic of both UC and Crohn's disease (1). This rupture in the IEB can result in inflammatory infiltrate resulting in a production of cytokines and other mediators that can further contribute to the impaired functioning of the IEB. Different proinflammatory cytokines are implicated in IEB dysfunction through the increased intestinal permeability along paracellular pathways (327). In particular cytokines such as TNF-α, IL-4, IL-13, interferon-γ (IFN-γ), IL-1β, IL-9, and IL-6 increase intestinal permeability, whereas has been shown that IL-10 has a protective role, maintain IEB function (321, 328–330). This is supported by the fact that IFN-γ and TNF-α are elevated in the mucosa of IBD patients contributing to a pro-inflammatory cascade and IEB disruption (75, 331).

Regarding the important role of genetic susceptibility in IBD patients, most genetic loci that confer susceptibility to Crohn's disease and UC, have been linked to defects in IEB function (332–335). The first Crohn's disease susceptibility gene identified was NOD2/CARD15 (336). It was demonstrated that patients with a mutation of NOD2 have altered cell-cell epithelial contacts (337). Furthermore, epithelial cells with mutated NOD2, have an inappropriate response to a sensor of a cell wall component of Gram-positive bacteria (muramyl dipeptide, MDP) whose stimulation determines the production of pro-inflammatory mediators (338–341). Moreover, it was shown that mutations in the NOD2 gene increased susceptibility to intestinal permeability in the healthy relatives compared to control subjects (342).

The intestinal microbiota has an important role as a regulator of epithelial–immune cell communication and patients with IBD often show dysbiosis (309). In fact in IBD have been documented changes in the commensal gut microbiota, represented by reduced complexity of commensals bacteria that are beneficial for the host or a greater representation of a specific phylum (343). Currently, it is not clear if these disorders are the cause or consequence of the manifestation of IBD. The cause of this condition seems to be disturbances in the recognition of pathogens microorganisms in the human intestine determined by alterations in the expression of pathogen recognition receptors, which include the already mentioned NOD2 (336, 340) and also TLRs and Rig-I like receptors. Intestinal epithelial cells (IECs) and innate immune cells, such as dendritic cells and macrophages, are equipped with these receptors to distinguish between components of pathogens and beneficial commensals (344). IECs have also the role to produce thymic stromal lymphopoietin (TSLP), IL-2 cytokine member, in response to commensals, such as Gram-negative *Escherichia coli* or Gram-positive *Lactobacillus rhamnosous* (345). Interestingly it was shown that in patients with Crohn's disease, IECs failed to produce TSLP, with consequent inability to control IL-12, produced by dendritic cells and involved in the development of Thelper 2 cells, resulting in alteration of intestinal homeostasis (346). These dysregulated epithelial-immune cell communication support the evidence of disturbed microbiota in patients with IBD (309).

Despite the involvement of immune intrinsic and barrier intrinsic dysfunction in the pathogenesis of IBD, none of these mechanisms alone would be able to explain all the characteristics of IBD. At the same time, data available in the literature, do not fully support defect in the IEB as a primary etiologic factor leading to the manifestation of IBD and it is unclear whether increased intestinal permeability is a consequence of the inflammatory process (347, 348) or anticipates and contributes to intestinal inflammation (317). Considering that current anti-inflammatory therapy (steroids and immunosuppressant drugs) remains unsatisfactory due to substantial side effects and uncontrolled relapses (349), understand the interaction between gut microbiota and IEB, particularly at the early subclinical phase of inflammatory disease, could be used to identify novel therapeutic approaches in chronic intestinal inflammation.

## Therapeutic Implications

Although several dietary factors, such as gluten, bile acids, fructose, ethanol, and emulsifiers are well-known agents damaging the IEB, other dietary components, such as fibers, SCFAs, glutamine, and vitamin D, may improve the IEB. Similarly, prebiotics, symbiotics, and probiotics may act on IEB function fortifying it. Recent extensive reviews have analyzed the effects of nutrients and supplements on IEB function (350, 351). Now, we will focus on nutritional approaches exerting a protective effect on the IEB, in particular on those that are better defined and studied such as glutamine, vitamin D, and SCFAs, particularly butyrate. Interestingly, these approaches have been proposed in the management of patients with IBS or IBD, conditions characterized by altered IEB function.

Glutamine is an essential amino acid in humans and one of the main energy sources for rapidly dividing epithelial cells of the gastrointestinal tract (352). Its depletion during illness or infection leads to intestinal epithelial cells atrophy resulting in increased intestinal permeability (352). Pioneer studies assessing the effect of enteral supplement with glutamine granules on intestinal IEB function in severely burned patients, showed that this compound can improve intestinal mucosal permeability, decrease plasma endotoxin levels, reduce hospital stay and costs (353, 354). A recent randomized controlled trial (RCT) assessing the efficacy and safety of oral glutamine supplementation in patients with post-infection IBS with diarrhea and increased intestinal permeability, showed that this treatment significantly improves all IBS symptoms and IEB function (355). However, due to the small sample size and the suboptimal experimental study design with significant methodological limitations, these results require caution and need to be replicated in larger well-performed clinical trials.

Vitamin D is a group of fat-soluble secosteroids responsible for increasing intestinal absorption of electrolytes including calcium, magnesium, and phosphate (356). Among the many other biological effects, vitamin D may activate the innate and modulate the adaptive immune systems with antibacterial, antiviral, and anti-inflammatory effects (357, 358). Vitamin D receptors are expressed by both epithelial and a large number of immune cells in the gastrointestinal tract (357, 358). Although *in vitro* studies showed that vitamin D is involved in the regulation of IEB function throughout the modulation of the expression of TJ molecules (357, 359, 360), few randomized controlled intervention studies have investigated its clinical efficacy or mechanisms of action in gastrointestinal diseases. Low levels of vitamin D are associated with IBD. A recent systematic review and meta-analysis of vitamin D therapy in patients with IBD and vitamin D deficiency has shown that this supplementation is effective not only for the correction of vitamin D levels but also for improving scores of clinical disease activity and biochemical markers (361). Although the mechanism of action is virtually unknown at this time, preliminary clinical data suggest that vitamin D supplementation in Crohn's disease patients in remission may have a prominent role in intestinal permeability maintenance over time (362).

SCFAs are essential molecules involved not only in host metabolism and immunity but also in IEB function (180, 363, 364). In addition, they are an energy source for intestinal cells and serve as signaling molecules with a beneficial role in intestinal homeostasis (180, 363, 364). Among SCFAs, butyrate is considered the most beneficial. Butyrate can be produced by a wide variety of bacteria and the most important butyrate-producing microorganisms in the human gut belong to the genera Faecalibacterium (in particular the species *Faecalibacterium prausnitzii*) and Roseburia (in particular the species *Roseburia intestinalis*) (365, 366). Sources for butyrate production include sugars, lactate, acetate, and amino acids, such as lysine (367). Although a lack of dietary fibers and SCFAs production can compromise both IEB integrity and mucus production, altering gut permeability, the role of SCFAs in DGBI pathophysiology is controversial (368). Similarly, although *in vitro* studies suggested that butyrate can improve paracellular permeability (183, 185), its role in restoring human intestinal permeability in clinical practice is very poorly defined. Preliminary data suggest that supplementation of sodium butyrate may improve IBS symptoms, particularly abdominal pain (369). In a recent proof-of-concept study performed in 40 patients with IBS, we showed that *Lactobacillus paracasei* CNCM I-1572 improves IBS symptoms through a significant reduction in genus *Ruminococcus* associated with a reduction of IL-15, linked with the modulation of IEB, and a significant increase in the fecal SCFAs acetate and butyrate (201). However, if nutritional butyrate exerts a protective effect on the IEB and may be effective in the management of patients with common gastrointestinal disorders, this should be demonstrated in *ad hoc* studies.

## Conclusions

IEB dysfunction is a common element of numerous intestinal and extra-intestinal diseases. Several factors can alter IEB including gut microbiota metabolites and immune system mediators. The intricate relationship among all these elements is still a matter of study. If IEB dysfunction is a cause or a consequence of the pathogenesis of common diseases including IBS, IBD, CD and the emerging NCGS is a challenge for the researcher. Solving this dilemma and deciphering the underlying molecular mechanisms will open the way to the development of new therapies and the optimization of the diagnostic process.

## Author Contributions

GB and VS designed the review. GB, MRB, DF, MP, FF, CC, and GM performed literature search and drafted the manuscript. All authors critically revised and approved the final version of the manuscript.

## Funding

This study was supported in part by the Italian Ministry of Education, University and Research and funds from the University of Bologna (RFO) to GB. GB was a recipient of the european grant HORIZON 2020-SC1-BHC-2018-2020/H2020-SC1-2019-Two-Stage-RTD-DISCOVERIE PROJECT. GB was a recipient of an educational grant from Fondazione del Monte di Bologna e Ravenna, and Fondazione Carisbo, Bologna, Italy. MRB was a recipient of a grant from the Italian Ministry of Health (Ricerca Finalizzata GR-2018-12367062). The funders had no role in study design, data collection and analysis, decision to publish, or preparation of the manuscript.

## Conflict of Interest

The authors declare that the research was conducted in the absence of any commercial or financial relationships that could be construed as a potential conflict of interest.

## Publisher's Note

All claims expressed in this article are solely those of the authors and do not necessarily represent those of their affiliated organizations, or those of the publisher, the editors and the reviewers. Any product that may be evaluated in this article, or claim that may be made by its manufacturer, is not guaranteed or endorsed by the publisher.

## Abbreviations

IEB: Intestinal epithelial barrier
GI: Gastrointestinal
TJs: tight junctions
IBS: irritable bowel syndrome
IBD: inflammatory bowel diseases
MUC2: mucin 2
AJs: adherens junctions
JAM-A: junctional adhesion molecule-A
ZO: zonula occludens
CD: celiac disease
NCGS: non-celiac gluten sensitivity
PAR-2: Proteinase-activated receptor 2
SCFAs: short-chain fatty acids
TLRs: Toll-like receptors
FGIDs: Functional Gastrointestinal Disorders
HLAs: human leucocyte antigens
GFD: gluten-free diet
UC: ulcerative colitis.

## References

[1] Turner JR. Intestinal mucosal barrier function in health and disease. Nat Rev Immunol. (2009) 9:799–809. 10.1038/nri2653 19855405

[2] Helander HF Fändriks L. Surface area of the digestive tract-revisited. Scand J Gastroenterol. (2014) 49:681–9. 10.3109/00365521.2014.898326 24694282

[3] Camilleri M. Leaky gut: mechanisms, measurement and clinical implications in humans. Gut. (2019) 68:1516–26. 10.1136/gutjnl-2019-318427 31076401 PMC6790068

[4] Yen TH Wright NA. The gastrointestinal tract stem cell niche. Stem Cell Rev. (2006) 2:203–12. 10.1007/s12015-006-0048-1 17625256

[5] Von Moltke J Ji M Liang HE Locksley RM. Tuft-cell-derived IL-25 regulates an intestinal ILC2-epithelial response circuit. Nature. (2016) 529:221–5. 10.1038/nature16161 26675736 PMC4830391

[6] Bischoff SC Barbara G Buurman W Ockhuizen T Schulzke JD Serino M. Intestinal permeability - a new target for disease prevention and therapy. BMC Gastroenterol. (2014) 14:189. 10.1186/s12876-014-0189-7 25407511 PMC4253991

[7] Salvo-Romero E Alonso-Cotoner C Pardo-Camacho C Casado-Bedmar M Vicario M. The intestinal barrier function and its involvement in digestive disease. Rev Esp Enfermedades Dig. (2015) 107:686–96. 10.17235/reed.2015.3846/2015 26541659

[8] Meddings J. The significance of the gut barrier in disease. Gut. (2008) 57:438–40. 10.1136/gut.2007.143172 18334657

[9] Hansson GC. Mucus and mucins in diseases of the intestinal and respiratory tracts. J Intern Med. (2019) 285:479–90. 10.1111/joim.12910 30963635 PMC6497544

[10] Gillois K Lévêque M Théodorou V Robert H Mercier-Bonin M. Mucus: an underestimated gut target for environmental pollutants and food additives. Microorganisms. (2018) 6:53. 10.3390/microorganisms6020053 29914144 PMC6027178

[11] Johansson MEV Hansson GC. Immunological aspects of intestinal mucus and mucins. Nat Rev Immunol. (2016) 16:639–49. 10.1038/nri.2016.88 27498766 PMC6435297

[12] Cone RA. Barrier properties of mucus. Adv Drug Deliv Rev. (2009) 61:75–85. 10.1016/j.addr.2008.09.008 19135107

[13] König J Wells J Cani PD García-Ródenas CL MacDonald T Mercenier A. Human intestinal barrier function in health and disease. Clin Transl Gastroenterol. (2016) 7:e196. 10.1038/ctg.2016.54 27763627 PMC5288588

[14] Kim YS Ho SB. Intestinal goblet cells and mucins in health and disease: recent insights and progress. Curr Gastroenterol Rep. (2010) 12:319–30. 10.1007/s11894-010-0131-2 20703838 PMC2933006

[15] Hansson GC. Mucins and the Microbiome. Annu Rev Biochem. (2020) 89:769–93. 10.1146/annurev-biochem-011520-105053 32243763 PMC8442341

[16] Bansil R Turner BS. The biology of mucus: composition, synthesis and organization. Adv Drug Deliv Rev. (2018) 124:3–15. 10.1016/j.addr.2017.09.023 28970050

[17] LAMONT JT. Mucus: the front line of intestinal mucosal defense. Ann N Y Acad Sci. (1992) 664:190–201. 10.1111/j.1749-6632.1992.tb39760.x 1456650

[18] Kim JJ Khan WI. Goblet cells and mucins: role in innate defense in enteric infections. Pathogens. (2013) 2:55–70. 10.3390/pathogens2010055 25436881 PMC4235714

[19] Strugnell RA Wijburg OLC. The role of secretory antibodies in infection immunity. Nat Rev Microbiol. (2010) 8:656–67. 10.1038/nrmicro2384 20694027

[20] Huus KE Petersen C Finlay BB. Diversity and dynamism of IgA–microbiota interactions. Nat Rev Immunol. (2021) 21:514–25. 10.1038/s41577-021-00506-1 33568782

[21] Pelaseyed T Hansson GC. Membrane mucins of the intestine at a glance. J Cell Sci. (2020) 133:jcs240929. 10.1242/JCS.240929 32169835 PMC7075048

[22] Etienne-Mesmin L Chassaing B Desvaux M De Paepe K Gresse R Sauvaitre T. Experimental models to study intestinal microbes–mucus interactions in health and disease. FEMS Microbiol Rev. (2019) 43:457–89. 10.1093/femsre/fuz013 31162610

[23] Pelaseyed T Bergström JH Gustafsson JK Ermund A Birchenough GMH Schütte A. The mucus and mucins of the goblet cells and enterocytes provide the first defense line of the gastrointestinal tract and interact with the immune system. Immunol Rev. (2014) 260:8–20. 10.1111/imr.12182 24942678 PMC4281373

[24] Pabst O Mowat AM. Oral tolerance to food protein. Mucosal Immunol. (2012) 5:232–9. 10.1038/mi.2012.4 22318493 PMC3328017

[25] Shan M Gentile M Yeiser JR Walland AC Bornstein VU Chen K. Mucus enhances gut homeostasis and oral tolerance by delivering immunoregulatory signals. Science. (2013) 342:447–53. 10.1126/science.1237910 24072822 PMC4005805

[26] Ermund A Gustafsson JK Hansson GC Keita Å V. Mucus properties and goblet cell quantification in mouse, rat and human ileal Peyer's patches. PLoS ONE. (2013) 8:e83688. 10.1371/journal.pone.0083688 24358305 PMC3865249

[27] Johansson MEV Holmén Larsson JM Hansson GC. The two mucus layers of colon are organized by the MUC2 mucin, whereas the outer layer is a legislator of host-microbial interactions. Proc Natl Acad Sci USA. (2011) 108:4659–65. 10.1073/pnas.1006451107 20615996 PMC3063600

[28] Atuma C Strugala V Allen A Holm L. The adherent gastrointestinal mucus gel layer: thickness and physical state *in vivo*. Am J Physiol Gastrointest Liver Physiol. (2001) 280:G922–9. 10.1152/ajpgi.2001.280.5.g922 11292601

[29] Ermund A Schütte A Johansson MEV Gustafsson JK Hansson GC. Studies of mucus in mouse stomach, small intestine, and colon. I. Gastrointestinal mucus layers have different properties depending on location as well as over the Peyer's patches. Am J Physiol Gastrointest Liver Physiol. (2013) 305:G341–7. 10.1152/ajpgi.00046.2013 23832518 PMC3761247

[30] Birchenough GMH Johansson MEV Gustafsson JK Bergström JH Hansson GC. New developments in goblet cell mucus secretion and function. Mucosal Immunol. (2015) 8:712–9. 10.1038/mi.2015.32 25872481 PMC4631840

[31] Ouellette AJ. Paneth cells and innate mucosal immunity. Curr Opin Gastroenterol. (2010) 26:547–53. 10.1097/MOG.0b013e32833dccde 20693892

[32] Heazlewood CK Cook MC Eri R Price GR Tauro SB Taupin D. Aberrant mucin assembly in mice causes endoplasmic reticulum stress and spontaneous inflammation resembling ulcerative colitis. PLoS Med. (2008) 5:54. 10.1371/journal.pmed.0050054 18318598 PMC2270292

[33] Renner M Bergmann G Krebs I End C Lyer S Hilberg F. DMBT1 confers mucosal protection *in vivo* and a deletion variant is associated with Crohn's disease. Gastroenterology. (2007) 133:1499–509. 10.1053/j.gastro.2007.08.007 17983803

[34] Hooper LV MacPherson AJ. Immune adaptations that maintain homeostasis with the intestinal microbiota. Nat Rev Immunol. (2010) 10:159–69. 10.1038/nri2710 20182457

[35] Meyer-Hoffert U Hornef MW Henriques-Normark B Axelsson LG Midtvedt T Pütsep K. Secreted enteric antimicrobial activity localises to the mucus surface layer. Gut. (2008) 57:764–71. 10.1136/gut.2007.141481 18250125

[36] Van Der Waaij LA Harmsen HJM Madjipour M Kroese FGM Zwiers M Van Dullemen HM. Bacterial population analysis of human colon and terminal ileum biopsies with 16S rRNA-based fluorescent probes: commensal bacteria live in suspension and have no direct contact with epithelial cells. Inflamm Bowel Dis. (2005) 11:865–71. 10.1097/01.mib.0000179212.80778.d3 16189415

[37] Johansson MEV Phillipson M Petersson J Velcich A Holm L Hansson GC. The inner of the two Muc2 mucin-dependent mucus layers in colon is devoid of bacteria. Proc Natl Acad Sci USA. (2008) 105:15064–9. 10.1073/pnas.0803124105 18806221 PMC2567493

[38] Johansson MEV Sjövall H Hansson GC. The gastrointestinal mucus system in health and disease. Nat Rev Gastroenterol Hepatol. (2013) 10:352–61. 10.1038/nrgastro.2013.35 23478383 PMC3758667

[39] Li H Limenitakis JP Fuhrer T Geuking MB Lawson MA Wyss M. The outer mucus layer hosts a distinct intestinal microbial niche. Nat Commun. (2015) 6:8292. 10.1038/ncomms9292 26392213 PMC4595636

[40] Kamphuis JBJ Mercier-Bonin M Eutamène H Theodorou V. Mucus organisation is shaped by colonic content; a new view. Sci Rep. (2017) 7:8527. 10.1038/s41598-017-08938-3 28819121 PMC5561085

[41] Hoskins LC Boulding ET. Mucin degradation in human colon ecosystems. J Clin Invest. (1981) 67:163–72. 10.1172/jci110009 6161136 PMC371584

[42] Png CW Lindén SK Gilshenan KS Zoetendal EG McSweeney CS Sly LI. Mucolytic bacteria with increased prevalence in IBD mucosa augment *in vitro* utilization of mucin by other bacteria. Am J Gastroenterol. (2010) 105:2420–8. 10.1038/ajg.2010.281 20648002

[43] Desai MS Seekatz AM Koropatkin NM Kamada N Hickey CA Wolter M. A dietary fiber-deprived gut microbiota degrades the colonic mucus barrier and enhances pathogen susceptibility. Cell. (2016) 167:1339–53.e21. 10.1016/j.cell.2016.10.043 27863247 PMC5131798

[44] Johansson MEV Jakobsson HE Holmén-Larsson J Schütte A Ermund A Rodríguez-Piñeiro AM. Normalization of host intestinal mucus layers requires long-term microbial colonization. Cell Host Microbe. (2015) 18:582–92. 10.1016/j.chom.2015.10.007 26526499 PMC4648652

[45] Schroeder BO. Fight them or feed them: how the intestinal mucus layer manages the gut microbiota. Gastroenterol Rep. (2019) 7:3–12. 10.1093/gastro/goy052 30792861 PMC6375348

[46] Fu J Wei B Wen T Johansson MEV Liu X Bradford E. Loss of intestinal core 1-derived O-glycans causes spontaneous colitis in mice. J Clin Invest. (2011) 121:1657–66. 10.1172/JCI45538 21383503 PMC3069788

[47] Larsson JMH Karlsson H Crespo JG Johansson MEV Eklund L Sjövall H. Altered O-glycosylation profile of MUC2 mucin occurs in active ulcerative colitis and is associated with increased inflammation. Inflamm Bowel Dis. (2011) 17:2299–307. 10.1002/ibd.21625 21290483

[48] Johansson MEV Gustafsson JK Holmen-Larsson J Jabbar KS Xia L Xu H. Bacteria penetrate the normally impenetrable inner colon mucus layer in both murine colitis models and patients with ulcerative colitis. Gut. (2014) 63:281–91. 10.1136/gutjnl-2012-303207 23426893 PMC3740207

[49] Strugala V Dettmar PW Pearson JP. Thickness and continuity of the adherent colonic mucus barrier in active and quiescent ulcerative colitis and Crohn's disease. Int J Clin Pract. (2008) 62:762–9. 10.1111/j.1742-1241.2007.01665.x 18194279

[50] Pullan RD Thomas GAO Rhodes M Newcombe RG Williams GT Allen A. Thickness of adherent mucus gel on colonic mucosa in humans and its relevance to colitis. Gut. (1994) 35:353–9. 10.1136/gut.35.3.353 8150346 PMC1374589

[51] Buisine MP Desreumaux P Leteurtre E Copin MC Colombel JF Porchet N. Mucin gene expression in intestinal epithelial cells in Crohn's disease. Gut. (2001) 49:544–51. 10.1136/gut.49.4.544 11559653 PMC1728475

[52] Buisine MP Desreumaux P Debailleul V Gambiez L Geboes K Ectors N. Abnormalities in mucin gene expression in Crohn's disease. Inflamm Bowel Dis. (1999) 5:24–32. 10.1097/00054725-199902000-00004 10028446

[53] Nakamori S Ota DM Cleary KR Shirotani K Irimura T. MUC1 mucin expression as a marker of progression and metastasis of human colorectal carcinoma. Gastroenterology. (1994) 106:353–61. 10.1016/0016-5085(94)90592-4 7905449

[54] Ajioka Y Allison LJ Jass JR. Significance of MUC1 and MUC2 mucin expression in colorectal cancer. J Clin Pathol. (1996) 49:560–4. 10.1136/jcp.49.7.560 8813954 PMC500570

[55] McGuckin MA Lindén SK Sutton P Florin TH. Mucin dynamics and enteric pathogens. Nat Rev Microbiol. (2011) 9:265–78. 10.1038/nrmicro2538 21407243

[56] Dharmani P Srivastava V Kissoon-Singh V Chadee K. Role of intestinal mucins in innate host defense mechanisms against pathogens. J Innate Immun. (2009) 1:123–35. 10.1159/000163037 20375571 PMC7312850

[57] Hayashi F Smith KD Ozinsky A Hawn TR Yi EC Goodlett DR. The innate immune response to bacterial flagellin is mediated by Toll-like receptor 5. Nature. (2001) 410:1099–103. 10.1038/35074106 11323673

[58] Birchenough GMH Nystrom EEL Johansson MEV Hansson GC. A sentinel goblet cell guards the colonic crypt by triggering Nlrp6-dependent Muc2 secretion. Science. (2016) 352:1535–42. 10.1126/science.aaf7419 27339979 PMC5148821

[59] Wang S Ahmadi S Nagpal R Jain S Mishra SP Kavanagh K. Lipoteichoic acid from the cell wall of a heat killed *Lactobacillus paracasei* D3-5 ameliorates aging-related leaky gut, inflammation and improves physical and cognitive functions: from *C. elegans* to mice. GeroScience. (2020) 42:333–52. 10.1007/s11357-019-00137-4 31814084 PMC7031475

[60] Lee KD Guk SM Chai JY. Toll-like receptor 2 and Muc2 expression on human intestinal epithelial cells by gymnophalloides seoi adult antigen. J Parasitol. (2010) 96:58–66. 10.1645/GE-2195.1 19737027

[61] Kamdar K Johnson AMF Chac D Myers K Kulur V Truevillian K. Innate recognition of the microbiota by TLR1 promotes epithelial homeostasis and prevents chronic inflammation. J Immunol. (2018) 201:230–42. 10.4049/jimmunol.1701216 29794015 PMC6903428

[62] Anderson JM Van Itallie CM. Physiology and function of the tight junction. Cold Spring Harb Perspect Biol. (2009) 1:a002584. 10.1101/cshperspect.a002584 20066090 PMC2742087

[63] Furuse M Fujita K Hiiragi T Fujimoto K Tsukita S. Claudin-1 and−2: novel integral membrane proteins localizing at tight junctions with no sequence similarity to occludin. J Cell Biol. (1998) 141:1539–50. 10.1083/jcb.141.7.1539 9647647 PMC2132999

[64] Furuse M Hirase T Itoh M Nagafuchi A Yonemura S Tsukita S. Occludin: a novel integral membrane protein localizing at tight junctions. J Cell Biol. (1993) 123:1777–88. 10.1083/jcb.123.6.1777 8276896 PMC2290891

[65] Martìn-Padura I Lostaglio S Schneemann M Williams L Romano M Fruscella P. Junctional adhesion molecule, a novel member of the immunoglobulin superfamily that distributes at intercellular junctions and modulates monocyte transmigration. J Cell Biol. (1998) 142:117–27. 10.1083/jcb.142.1.117 9660867 PMC2133024

[66] Ikenouchi J Umeda K Tsukita S Furuse M Tsukita S. Requirement of ZO-1 for the formation of belt-like adherens junctions during epithelial cell polarization. J Cell Biol. (2007) 176:779–86. 10.1083/jcb.200612080 17353356 PMC2064052

[67] Günzel D Fromm M. Claudins and other tight junction proteins. Compr Physiol. (2012) 2:1819–52. 10.1002/cphy.c110045 23723025

[68] Günzel D Yu ASL. Claudins and the modulation of tight junction permeability. Physiol Rev. (2013) 93:525–69. 10.1152/physrev.00019.2012 23589827 PMC3768107

[69] Suzuki T. Regulation of the intestinal barrier by nutrients: the role of tight junctions. Anim Sci J. (2020) 91:e13357. 10.1111/asj.13357 32219956 PMC7187240

[70] Cong X Kong W. Endothelial tight junctions and their regulatory signaling pathways in vascular homeostasis and disease. Cell Signal. (2020) 66:109485. 10.1016/j.cellsig.2019.109485 31770579

[71] Shen L Weber CR Raleigh DR Yu D Turner JR. Tight junction pore and leak pathways: a dynamic duo. Annu Rev Physiol. (2011) 73:283–309. 10.1146/annurev-physiol-012110-142150 20936941 PMC4655434

[72] Van Itallie CM Anderson JM. Claudins and epithelial paracellular transport. Annu Rev Physiol. (2006) 68:403–29. 10.1146/annurev.physiol.68.040104.131404 16460278

[73] Heller F Florian P Bojarski C Richter J Christ M Hillenbrand B. Interleukin-13 is the key effector Th2 cytokine in ulcerative colitis that affects epithelial tight junctions, apoptosis, and cell restitution. Gastroenterology. (2005) 129:550–64. 10.1016/j.gastro.2005.05.002 16083712

[74] Ivanov AI Nusrat A Parkos CA. The epithelium in inflammatory bowel disease: potential role of endocytosis of junctional proteins in barrier disruption. Novartis Found Symp. (2004) 263:115–24. 10.1002/0470090480.ch9 15669638

[75] Kucharzik T Walsh S V. Chen J Parkos CA Nusrat A. Neutrophil transmigration in inflammatory bowel disease is associated with differential expression of epithelial intercellular junction proteins. Am J Pathol. (2001) 159:2001–9. 10.1016/S0002-9440(10)63051-9 11733350 PMC1850599

[76] Pizzuti D Senzolo M Buda A Chiarelli S Giacomelli L Mazzon E. *In vitro* model for IgE mediated food allergy. Scand J Gastroenterol. (2011) 46:177–87. 10.3109/00365521.2010.525716 21028948

[77] Assimakopoulos SF Tsamandas AC Tsiaoussis GI Karatza E Triantos C Vagianos CE. Altered intestinal tight junctions' expression in patients with liver cirrhosis: a pathogenetic mechanism of intestinal hyperpermeability. Eur J Clin Invest. (2012) 42:439–46. 10.1111/j.1365-2362.2011.02609.x 22023490

[78] Bertiaux-Vandaële N Youmba SB Belmonte L Lecleire S Antonietti M Gourcerol G. The expression and the cellular distribution of the tight junction proteins are altered in irritable bowel syndrome patients with differences according to the disease subtype. Am J Gastroenterol. (2011) 106:2165–73. 10.1038/ajg.2011.257 22008894

[79] Rahner C Mitic LL Anderson JM. Heterogeneity in expression and subcellular localization of claudins 2, 3, 4, and 5 in the rat liver, pancreas, and gut. Gastroenterology. (2001) 120:411–22. 10.1053/gast.2001.21736 11159882

[80] Reyes JL Lamas M Martin D Namorado MDC Islas S Luna J. The renal segmental distribution of claudins changes with development. Kidney Int. (2002) 62:476–87. 10.1046/j.1523-1755.2002.00479.x 12110008

[81] Wolburg H Wolburg-Buchholz K Liebner S Engelhardt B. Claudin-1, claudin-2 and claudin-11 are present in tight junctions of choroid plexus epithelium of the mouse. Neurosci Lett. (2001) 307:77–80. 10.1016/S0304-3940(01)01927-9 11427304

[82] Zhu Y Brännström M Janson PO Sundfeldt K. Differences in expression patterns of the tight junction proteins, claudin 1, 3, 4 and 5, in human ovarian surface epithelium as compared to epithelia in inclusion cysts and epithelial ovarian tumours. Int J Cancer. (2006) 118:1884–91. 10.1002/ijc.21506 16287068

[83] Oshima T Miwa H Joh T. Changes in the expression of claudins in active ulcerative colitis. J Gastroenterol Hepatol. (2008) 23:3–7. 10.1111/j.1440-1746.2008.05405.x 19120888

[84] Nagy Szakál D Gyorffy H Arató A Cseh Á Molnár K Papp M. Mucosal expression of claudins 2, 3 and 4 in proximal and distal part of duodenum in children with coeliac disease. Virchows Arch. (2010) 456:245–50. 10.1007/s00428-009-0879-7 20143085

[85] Martínez C Lobo B Pigrau M Ramos L González-Castro AM Alonso C. Diarrhoea-predominant irritable bowel syndrome: an organic disorder with structural abnormalities in the jejunal epithelial barrier. Gut. (2013) 62:1160–8. 10.1136/gutjnl-2012-302093 22637702

[86] Zeissig S Bürgel N Günzel D Richter J Mankertz J Wahnschaffe U. Changes in expression and distribution of claudin 2, 5 and 8 lead to discontinuous tight junctions and barrier dysfunction in active Crohn's disease. Gut. (2007) 56:61–72. 10.1136/gut.2006.094375 16822808 PMC1856677

[87] Laurila JJ Karttunen T Koivukangas V Laurila PA Syrjälä H Saarnio J. Tight junction proteins in gallbladder epithelium: different expression in acute acalculous and calculous cholecystitis. J Histochem Cytochem. (2007) 55:567–73. 10.1369/jhc.6A7155.2007 17283368

[88] Wolburg H Wolburg-Buchholz K Kraus J Rascher-Eggstein G Liebner S Hamm S. Localization of claudin-3 in tight junctions of the blood-brain barrier is selectively lost during experimental autoimmune encephalomyelitis and human glioblastoma multiforme. Acta Neuropathol. (2003) 105:586–92. 10.1007/s00401-003-0688-z 12734665

[89] Kiuchi-Saishin Y Gotoh S Furuse M Takasuga A Tano Y Tsukita S. Differential expression patterns of claudins, tight junction membrane proteins, in mouse nephron segments. J Am Soc Nephrol. (2002) 13:875–86. 10.1681/asn.v134875 11912246

[90] Mennigen R Nolte K Rijcken E Utech M Loeffler B Senninger N. Probiotic mixture VSL#3 protects the epithelial barrier by maintaining tight junction protein expression and preventing apoptosis in a murine model of colitis. Am J Physiol Gastrointest Liver Physiol. (2009) 296:G1140–9. 10.1152/ajpgi.90534.2008 19221015

[91] Oshima T Miwa H. Gastrointestinal mucosal barrier function and diseases. J Gastroenterol. (2016) 51:768–78. 10.1007/s00535-016-1207-z 27048502

[92] Sapone A Lammers KM Casolaro V Cammarota M Giuliano MT De Rosa M. Divergence of gut permeability and mucosal immune gene expression in two gluten-associated conditions: Celiac disease and gluten sensitivity. BMC Med. (2011) 9:23. 10.1186/1741-7015-9-23 21392369 PMC3065425

[93] Morita K Sasaki H Furuse M Tsukita S. Endothelial claudin: Claudin-5/TMVCF constitutes tight junction strands in endothelial cells. J Cell Biol. (1999) 147:185–94. 10.1083/jcb.147.1.185 10508865 PMC2164984

[94] Nitta T Hata M Gotoh S Seo Y Sasaki H Hashimoto N. Size-selective loosening of the blood-brain barrier in claudin-5-deficient mice. J Cell Biol. (2003) 161:653–60. 10.1083/jcb.200302070 12743111 PMC2172943

[95] Amasheh S Schmidt T Mahn M Florian P Mankertz J Tavalali S. Contribution of claudin-5 to barrier properties in tight junctions of epithelial cells. Cell Tissue Res. (2005) 321:89–96. 10.1007/s00441-005-1101-0 16158492

[96] Schumann M Günzel D Buergel N Richter JF Troeger H May C. Cell polarity-determining proteins Par-3 and PP-1 are involved in epithelial tight junction defects in coeliac disease. Gut. (2012) 61:220–8. 10.1136/gutjnl-2011-300123 21865402

[97] Fujita H Chiba H Yokozaki H Sakai N Sugimoto K Wada T. Differential expression and subcellular localization of claudin-7,−8,−12,−13, and−15 along the mouse intestine. J Histochem Cytochem. (2006) 54:933–44. 10.1369/jhc.6A6944.2006 16651389

[98] Go M Kojima T Takano KI Murata M Ichimiya S Tsubota H. Expression and function of tight junctions in the crypt epithelium of human palatine tonsils. J Histochem Cytochem. (2004) 52:1627–38. 10.1369/jhc.4A6339.2004 15557217

[99] Li WY Huey CL Yu AS. Expression of claudin-7 and−8 along the mouse nephron. Am J Physiol Renal Physiol. (2004) 286:F1063–71. 10.1152/ajprenal.00384.2003 14722018

[100] Turksen K Troy TC. Claudin-6: a novel tight junction molecule is developmentally regulated in mouse embryonic epithelium. Dev Dyn. (2001) 222:292–300. 10.1002/dvdy.1174 11668606

[101] Lameris AL Huybers S Kaukinen K Mäkelä TH Bindels RJ Hoenderop JG. Expression profiling of claudins in the human gastrointestinal tract in health and during inflammatory bowel disease. Scand J Gastroenterol. (2013) 48:58–69. 10.3109/00365521.2012.741616 23205909

[102] Niimi T Nagashima K Ward JM Minoo P Zimonjic DB Popescu NC. claudin-18, a novel downstream target gene for the T/EBP/NKX2.1 homeodomain transcription factor, encodes lung- and stomach-specific isoforms through alternative splicing. Mol Cell Biol. (2001) 21:7380–90. 10.1128/mcb.21.21.7380-7390.2001 11585919 PMC99911

[103] Linares GR Brommage R Powell DR Xing W Chen ST Alshbool FZ. Claudin 18 is a novel negative regulator of bone resorption and osteoclast differentiation. J Bone Miner Res. (2012) 27:1553–65. 10.1002/jbmr.1600 22437732 PMC3377820

[104] Sanada Y Oue N Mitani Y Yoshida K Nakayama H Yasui W. Down-regulation of the claudin-18 gene, identified through serial analysis of gene expression data analysis, in gastric cancer with an intestinal phenotype. J Pathol. (2006) 208:633–42. 10.1002/path.1922 16435283

[105] Wong V. Phosphorylation of occludin correlates with occludin localization and function at the tight junction. Am J Physiol Cell Physiol. (1997) 273:C1859–67. 10.1152/ajpcell.1997.273.6.c1859 9435490

[106] Umeda K Ikenouchi J Katahira-Tayama S Furuse K Sasaki H Nakayama M. ZO-1 and ZO-2 independently determine where claudins are polymerized in tight-junction strand formation. Cell. (2006) 126:741–54. 10.1016/j.cell.2006.06.043 16923393

[107] González-Mariscal L Quirós M Díaz-Coránguez M. ZO proteins and redox-dependent processes. Antioxidants Redox Signal. (2011) 15:1235–53. 10.1089/ars.2011.3913 21294657

[108] Jesaitis LA Goodenough DA. Molecular characterization and tissue distribution of ZO-2, a tight junction protein homologous to ZO-1 and the *Drosophila* discs-large tumor suppressor protein. J Cell Biol. (1994) 124:949–61. 10.1083/jcb.124.6.949 8132716 PMC2119984

[109] Fanning AS Jameson BJ Jesaitis LA Anderson JM. The tight junction protein ZO-1 establishes a link between the transmembrane protein occludin and the actin cytoskeleton. J Biol Chem. (1998) 273:29745–53. 10.1074/jbc.273.45.29745 9792688

[110] Itoh M Morita K Tsukita S. Characterization of ZO-2 as a MAGUK family member associated with tight as well as adherens junctions with a binding affinity to occludin and α catenin. J Biol Chem. (1999) 274:5981–6. 10.1074/jbc.274.9.5981 10026224

[111] Fanning AS Ma TY Anderson JM. Isolation and functional characterization of the actin binding region in the tight junction protein ZO-1. FASEB J. (2002) 16:1835–7. 10.1096/fj.02-0121fje 12354695

[112] Mandell KJ Parkos CA. The JAM family of proteins. Adv Drug Deliv Rev. (2005) 57:857–67. 10.1016/j.addr.2005.01.005 15820556

[113] Ebnet K. Junctional adhesion molecules (JAMs): cell adhesion receptors with pleiotropic functions in cell physiology and development. Physiol Rev. (2017) 97:1529–54. 10.1152/physrev.00004.2017 28931565

[114] Van Itallie CM Anderson JM. Architecture of tight junctions and principles of molecular composition. Semin Cell Dev Biol. (2014) 36:157–65. 10.1016/j.semcdb.2014.08.011 25171873 PMC4254347

[115] Laukoetter MG Nava P Lee WY Severson EA Capaldo CT Babbin BA. JAM-A regulates permeability and inflammation in the intestine *in vivo*. J Exp Med. (2007) 204:3067–76. 10.1084/jem.20071416 18039951 PMC2150975

[116] Severson EA Lee WY Capaldo CT Nusrat A Parkos CA. Junctional adhesion molecule a interacts with afadin and PDZ-GEF2 to activate raplA, regulate j31 integrin levels, and enhance cell migration. Mol Biol Cell. (2009) 20:1916–25. 10.1091/mbc.E08-10-1014 19176753 PMC2663925

[117] Nava P Capaldo CT Koch S Kolegraff K Rankin CR Farkas AE. JAM-A regulates epithelial proliferation through Akt/β-catenin signalling. EMBO Rep. (2011) 12:314–20. 10.1038/embor.2011.16 21372850 PMC3077244

[118] Monteiro AC Sumagin R Rankin CR Leoni G Mina MJ Reiter DM. JAM-A associates with ZO-2, afadin, and PDZ-GEF1 to activate Rap2c and regulate epithelial barrier function. Mol Biol Cell. (2013) 24:2849–60. 10.1091/mbc.E13-06-0298 23885123 PMC3771947

[119] Hollander D Vadheim CM Brettholz E Petersen GM Delahunty T Rotter JI. Increased intestinal permeability in patients with Crohn's disease and their relatives: a possible etiologic factor. Ann Intern Med. (1986) 105:883–5. 10.7326/0003-4819-105-6-883 3777713

[120] Martínez C Vicario M Ramos L Lobo B Mosquera JL Alonso C. The jejunum of diarrhea-predominant irritable bowel syndrome shows molecular alterations in the tight junction signaling pathway that are associated with mucosal pathobiology and clinical manifestations. Am J Gastroenterol. (2012) 107:736–46. 10.1038/ajg.2011.472 22415197

[121] Wilcz-Villega E Mcclean S O'Sullivan M. Reduced E-cadherin expression is associated with abdominal pain and symptom duration in a study of alternating and diarrhea predominant IBS. Neurogastroenterol Motil. (2014) 26:316–25. 10.1111/nmo.12262 24286617

[122] Drago S El Asmar R Di Pierro M Clemente MG Tripathi A Sapone A. Gliadin, zonulin and gut permeability: effects on celiac and non-celiac intestinal mucosa and intestinal cell lines. Scand J Gastroenterol. (2006) 41:408–19. 10.1080/00365520500235334 16635908

[123] Vetrano S Rescigno M Rosaria Cera M Correale C Rumio C Doni A. Unique role of junctional adhesion molecule-a in maintaining mucosal homeostasis in inflammatory Bowel disease. Gastroenterology. (2008) 135:173–84. 10.1053/j.gastro.2008.04.002 18514073

[124] Wilcz-Villega EM McClean S O'Sullivan MA. Mast cell tryptase reduces junctional adhesion molecule-A (JAM-A) expression in intestinal epithelial cells: implications for the mechanisms of barrier dysfunction in irritable bowel syndrome. Am J Gastroenterol. (2013) 108:1140–51. 10.1038/ajg.2013.92 23588236

[125] Cordenonsi M D'Atri F Hammar E Parry DAD Kendrick-Jones J Shore D. Cingulin contains globular and coiled-coil domains and interacts with ZO-1, ZO-2, ZO-3, and myosin. J Cell Biol. (1999) 147:1569–81. 10.1083/jcb.147.7.1569 10613913 PMC2174252

[126] Citi S Paschoud S Pulimeno P Timolati F De Robertis F Jond L. The tight junction protein cingulin regulates gene expression and rhoA signaling. Ann N Y Acad Sci. (2009) 1165:88–98. 10.1111/j.1749-6632.2009.04053.x 19538293

[127] Suarez C Kovar DR. Internetwork competition for monomers governs actin cytoskeleton organization. Nat Rev Mol Cell Biol. (2016) 17:799–810. 10.1038/nrm.2016.106 27625321 PMC5125073

[128] Kim S Coulombe PA. Emerging role for the cytoskeleton as an organizer and regulator of translation. Nat Rev Mol Cell Biol. (2010) 11:75–81. 10.1038/nrm2818 20027187

[129] Al-Sadi RM Ma TY. IL-1β Causes an Increase in Intestinal Epithelial Tight Junction Permeability. J Immunol. (2007) 178:4641–9. 10.4049/jimmunol.178.7.4641 17372023 PMC3724221

[130] Schwayer C Shamipour S Pranjic-Ferscha K Schauer A Balda M Tada M. Mechanosensation of tight junctions depends on ZO-1 phase separation and flow. Cell. (2019) 179:937–52.e18. 10.1016/j.cell.2019.10.006 31675500

[131] Holthöfer B Windoffer R Troyanovsky S Leube RE. Structure and function of desmosomes. Int Rev Cytol. (2007) 264:65–163. 10.1016/S0074-7696(07)64003-0 17964922

[132] Hartsock A Nelson WJ. Adherens and tight junctions: structure, function and connections to the actin cytoskeleton. Biochim Biophys Acta Biomembr. (2008) 1778:660–9. 10.1016/j.bbamem.2007.07.012 17854762 PMC2682436

[133] Shapiro L Weis WI. Structure and biochemistry of cadherins and catenins. Cold Spring Harb Perspect Biol. (2009) 1:a003053. 10.1101/cshperspect.a003053 20066110 PMC2773639

[134] Ivanov AI Naydenov NG. Dynamics and regulation of epithelial adherens junctions. Recent discoveries and controversies. Int Rev Cell Mol Biol. (2013) 303:27–99. 10.1016/B978-0-12-407697-6.00002-7 23445808

[135] Takeichi M. Dynamic contacts: rearranging adherens junctions to drive epithelial remodelling. Nat Rev Mol Cell Biol. (2014) 15:397–410. 10.1038/nrm3802 24824068

[136] Nekrasova OE Amargo EV Smith WO Chen J Kreitzer GE Green KJ. Desmosomal cadherins utilize distinct kinesins for assembly into desmosomes. J Cell Biol. (2011) 195:1185–203. 10.1083/jcb.201106057 22184201 PMC3246898

[137] Hatzfeld M Keil R Magin TM. Desmosomes and intermediate filaments: their consequences for tissue mechanics. Cold Spring Harb Perspect Biol. (2017) 9:a029157. 10.1101/cshperspect.a029157 28096266 PMC5453391

[138] Tripathi A Lammers KM Goldblum S Shea-Donohue T Netzel-Arnett S Buzza MS. Identification of human zonulin, a physiological modulator of tight junctions, as prehaptoglobin-2. Proc Natl Acad Sci USA. (2009) 106:16799–804. 10.1073/pnas.0906773106 19805376 PMC2744629

[139] Lammers KM Lu R Brownley J Lu B Gerard C Thomas K. Gliadin induces an increase in intestinal permeability and zonulin release by binding to the chemokine receptor CXCR3. Gastroenterology. (2008) 135:194–204.e3. 10.1053/j.gastro.2008.03.023 18485912 PMC2653457

[140] Sapone A De Magistris L Pietzak M Clemente MG Tripathi A Cucca F. Zonulin upregulation is associated with increased gut permeability in subjects with type 1 diabetes and their relatives. Diabetes. (2006) 55:1443–9. 10.2337/db05-1593 16644703

[141] Barbaro MR Cremon C Wrona D Fuschi D Marasco G Stanghellini V. Non-celiac gluten sensitivity in the context of functional gastrointestinal disorders. Nutrients. (2020) 12:1–21. 10.3390/nu12123735 33291590 PMC7761787

[142] Fasano A. Zonulin measurement conundrum: add confusion to confusion does not lead to clarity. Gut. (2020) 70:2007–8. 10.1136/gutjnl-2020-323367 33177164

[143] Misra A. Challenges in delivery of therapeutic genomics and proteomics. Amsterdam: Elsevier Inc. (2011). 10.1016/C2010-0-65663-X

[144] Sugano K Kansy M Artursson P Avdeef A Bendels S Di L. Coexistence of passive and carrier-mediated processes in drug transport. Nat Rev Drug Discov. (2010) 9:597–614. 10.1038/nrd3187 20671764

[145] Wang Y DeMazumder D Hill JA. Ionic fluxes and genesis of the cardiac action potential. Muscle. (2012) 1:67–85. 10.1016/B978-0-12-381510-1.00007-7

[146] Horisberger JD Chraïbi A. Epithelial sodium channel: a ligand-gated channel? Nephron Physiol. (2004) 96:37–41. 10.1159/000076406 14988660

[147] Mukherjee B Satapathy BS Bhattacharya S Chakraborty R Mishra VP. Chapter 19 - Pharmacokinetic and pharmacodynamic modulations of therapeutically active constituents from orally administered nanocarriers along with a glimpse of their advantages and limitations. In: Grumezescu AM, editor. Nano- and Microscale Drug Delivery Systems. Elsevier. (2017). p. 357–75. 10.1016/B978-0-323-52727-9.00019-4

[148] Goldstein JL Anderson RGW Brown MS. Coated pits, coated vesicles, and receptor-mediated endocytosis. Nature. (1979) 279:679–85. 10.1038/279679a0 221835

[149] Garcia-Castillo MD Chinnapen DJF Lencer WI. Membrane transport across polarized epithelia. Cold Spring Harb Perspect Biol. (2017) 9:a027912. 10.1101/cshperspect.a027912 28213463 PMC5585844

[150] Sandvig K Kavaliauskiene S Skotland T. Clathrin-independent endocytosis: an increasing degree of complexity. Histochem Cell Biol. (2018) 150:107–18. 10.1007/s00418-018-1678-5 29774430 PMC6096564

[151] Tuma PL Hubbard AL. Transcytosis: crossing cellular barriers. Physiol Rev. (2003) 83:871–932. 10.1152/physrev.00001.2003 12843411

[152] Mestecky J Russell MW Elson CO. Intestinal IgA: novel views on its function in the defence of the largest mucosal surface. Gut. (1999) 44:2–5. 10.1136/gut.44.1.2 9862815 PMC1760065

[153] Kadaoui KA Corthésy B. Secretory IgA mediates bacterial translocation to dendritic cells in mouse Peyer's patches with restriction to mucosal compartment. J Immunol. (2007) 179:7751–7. 10.4049/jimmunol.179.11.7751 18025221

[154] Boullier S Tanguy M Kadaoui KA Caubet C Sansonetti P Corthésy B. Secretory IgA-mediated neutralization of *Shigella flexneri* prevents intestinal tissue destruction by down-regulating inflammatory circuits. J Immunol. (2009) 183:5879–85. 10.4049/jimmunol.0901838 19828639

[155] Rey J Garin N Spertini F Corthésy B. Targeting of secretory IgA to Peyer's patch dendritic and T cells after transport by intestinal M cells. J Immunol. (2004) 172:3026–33. 10.4049/jimmunol.172.5.3026 14978107

[156] Matysiak-Budnik T Moura IC Arcos-Fajardo M Lebreton C Ménard S Candalh C. Secretory IgA mediates retrotranscytosis of intact gliadin peptides via the transferrin receptor in celiac disease. J Exp Med. (2008) 205:143–54. 10.1084/jem.20071204 18166587 PMC2234361

[157] Bevilacqua C Montagnac G Benmerah A Candalh C Brousse N Cerf-Bensussan N. Food allergens are protected from degradation during CD23-mediated transepithelial transport. Int Arch Allergy Immunol. (2004) 205:143–54. 10.1159/000080653 15345909

[158] Kaiserlian D Lachaux A Grosjean I Graber P Bonnefoy JY. Intestinal epithelial cells express the CD23/FcεRII molecule: enhanced expression in enteropathies. Immunology. (1993) 80:90–5. 8244467 PMC1422110

[159] Montagnac G Yu LCH Bevilacqua C Heyman M Conrad DH Perdue MH. Differential role for CD23 splice forms in apical to basolateral transcytosis of IgE/allergen complexes. Traffic. (2005) 6:230–42. 10.1111/j.1600-0854.2005.00262.x 15702991

[160] Montagnac G Mollà-Herman A Bouchet J Yu LCH Conrad DH Perdue MH. Intracellular trafficking of CD23: differential regulation in humans and mice by both extracellular and intracellular exons. J Immunol. (2005) 174:5562–72. 10.4049/jimmunol.174.9.5562 15843555

[161] Neal MD Leaphart C Levy R Prince J Billiar TR Watkins S. Enterocyte TLR4 Mediates Phagocytosis and Translocation of Bacteria Across the Intestinal Barrier. J Immunol. (2006) 176:3070–9. 10.4049/jimmunol.176.5.3070 16493066

[162] Conner SD Schmid SL. Regulated portals of entry into the cell. Nature. (2003) 422:37–44. 10.1038/nature01451 12621426

[163] Günther J Seyfert HM. The first line of defence: insights into mechanisms and relevance of phagocytosis in epithelial cells. Semin Immunopathol. (2018) 40:555–65. 10.1007/s00281-018-0701-1 30182191 PMC6223882

[164] Hommelgaard AM Roepstorff K Vilhardt F Torgersen ML Sandvig K van Deurs B. Caveolae: stable membrane domains with a potential for internalization. Traffic. (2005) 6:720–4. 10.1111/j.1600-0854.2005.00314.x 16101676

[165] Maloy KJ Powrie F. Intestinal homeostasis and its breakdown in inflammatory bowel disease. Nature. (2011) 474:298–306. 10.1038/nature10208 21677746

[166] Khor B Gardet A Xavier RJ. Genetics and pathogenesis of inflammatory bowel disease. Nature. (2011) 474:307–17. 10.1038/nature10209 21677747 PMC3204665

[167] Suzuki T. Regulation of intestinal epithelial permeability by tight junctions. Cell Mol Life Sci. (2013) 70:631–59. 10.1007/s00018-012-1070-x 22782113 PMC11113843

[168] Berkes J Viswanathan VK Savkovic SD Hecht G. Intestinal epithelial responses to enteric pathogens: effects on the tight junction barrier, ion transport, and inflammation. Gut. (2003) 52:439–51. 10.1136/gut.52.3.439 12584232 PMC1773546

[169] Bäckhed F Ley RE Sonnenburg JL Peterson DA Gordon JI. Host-bacterial mutualism in the human intestine. Science. (2005) 307:1915–20. 10.1126/science.1104816 15790844

[170] Hooper L V. Littman DR Macpherson AJ. Interactions between the microbiota and the immune system. Science. (2012) 336:1268–73. 10.1126/science.1223490 22674334 PMC4420145

[171] Kayama H Okumura R Takeda K. Interaction between the microbiota, epithelia, and immune cells in the intestine. Annu Rev Immunol. (2020) 38:23–48. 10.1146/annurev-immunol-070119-115104 32340570

[172] Sonnenburg ED Smits SA Tikhonov M Higginbottom SK Wingreen NS Sonnenburg JL. Diet-induced extinctions in the gut microbiota compound over generations. Nature. (2016) 529:212–5. 10.1038/nature16504 26762459 PMC4850918

[173] LeBlanc JG Milani C de Giori GS Sesma F van Sinderen D Ventura M. Bacteria as vitamin suppliers to their host: a gut microbiota perspective. Curr Opin Biotechnol. (2013) 24:160–8. 10.1016/j.copbio.2012.08.005 22940212

[174] Baümler AJ Sperandio V. Interactions between the microbiota and pathogenic bacteria in the gut. Nature. (2016) 535:85–93. 10.1038/nature18849 27383983 PMC5114849

[175] Buffie CG Pamer EG. Microbiota-mediated colonization resistance against intestinal pathogens. Nat Rev Immunol. (2013) 13:790–801. 10.1038/nri3535 24096337 PMC4194195

[176] Gensollen T Iyer SS Kasper DL Blumberg RS. How colonization by microbiota in early life shapes the immune system. Science. (2016) 352:539–44. 10.1126/science.aad9378 27126036 PMC5050524

[177] Thaiss CA Zmora N Levy M Elinav E. The microbiome and innate immunity. Nature. (2016) 535:65–74. 10.1038/nature18847 27383981

[178] Stecher B Hardt WD. Mechanisms controlling pathogen colonization of the gut. Curr Opin Microbiol. (2011) 14:82–91. 10.1016/j.mib.2010.10.003 21036098

[179] Keeney KM Finlay BB. Enteric pathogen exploitation of the microbiota-generated nutrient environment of the gut. Curr Opin Microbiol. (2011) 14:92–8. 10.1016/j.mib.2010.12.012 21215681 PMC3039043

[180] Litvak Y Byndloss MX Bäumler AJ. Colonocyte metabolism shapes the gut microbiota. Science. (2018) 362:eaat9076. 10.1126/science.aat9076 30498100 PMC6296223

[181] van Thiel IAM de Jonge WJ Chiu IM van den Wijngaard RM. Microbiota-neuroimmune cross talk in stress-induced visceral hypersensitivity of the bowel. Am J Physiol Gastrointest Liver Physiol. (2020) 318:G1034–41. 10.1152/ajpgi.00196.2019 32308040 PMC7642838

[182] Chowdhury SR King DE Willing BP Band MR Beever JE Lane AB. Transcriptome profiling of the small intestinal epithelium in germfree versus conventional piglets. BMC Genomics. (2007) 8:215. 10.1186/1471-2164-8-215 17615075 PMC1949829

[183] Burger-van Paassen N Vincent A Puiman PJ van der Sluis M Bouma J Boehm G. The regulation of intestinal mucin MUC2 expression by short-chain fatty acids: implications for epithelial protection. Biochem J. (2009) 420:211–9. 10.1042/BJ20082222 19228118

[184] Kim MH Kang SG Park JH Yanagisawa M Kim CH. Short-chain fatty acids activate GPR41 and GPR43 on intestinal epithelial cells to promote inflammatory responses in mice. Gastroenterology. (2013) 145:396–406.e1-10. 10.1053/j.gastro.2013.04.056 23665276

[185] Singh N Gurav A Sivaprakasam S Brady E Padia R Shi H. Activation of Gpr109a, receptor for niacin and the commensal metabolite butyrate, suppresses colonic inflammation and carcinogenesis. Immunity. (2014) 40:128–39. 10.1016/j.immuni.2013.12.007 24412617 PMC4305274

[186] Ghosh S Whitley CS Haribabu B Jala VR. Regulation of intestinal barrier function by microbial. Cell Mol Gastroenterol Hepatol. (2021) 11:1463–82. 10.1016/j.jcmgh.2021.02.007 33610769 PMC8025057

[187] Abreu MT. Toll-like receptor signalling in the intestinal epithelium: how bacterial recognition shapes intestinal function. Nat Rev Immunol. (2010) 10:131–43. 10.1038/nri2707 20098461

[188] Burgueño JF Abreu MT. Epithelial Toll-like receptors and their role in gut homeostasis and disease. Nat Rev Gastroenterol Hepatol. (2020) 17:263–78. 10.1038/s41575-019-0261-4 32103203

[189] Allam-Ndoul B Castonguay-Paradis S Veilleux A. Gut microbiota and intestinal trans-epithelial permeability. Int J Mol Sci. (2020) 21:1–14. 10.3390/ijms21176402 32899147 PMC7503654

[190] Hayes CL Dong J Galipeau HJ Jury J McCarville J Huang X. Commensal microbiota induces colonic barrier structure and functions that contribute to homeostasis. Sci Rep. (2018) 8:14184. 10.1038/s41598-018-32366-6 30242285 PMC6155058

[191] Hooper L V. Wong MH Thelin A Hansson L Falk PG Gordon JI. Molecular analysis of commensal host-microbial relationships in the intestine. Science. (2001) 291:881–4. 10.1126/science.291.5505.881 11157169

[192] Ukena SN Singh A Dringenberg U Engelhardt R Seidler U Hansen W. Probiotic *Escherichia coli* Nissle 1917 inhibits leaky gut by enhancing mucosal integrity. PLoS ONE. (2007) 2:e1308. 10.1371/journal.pone.0001308 18074031 PMC2110898

[193] Barbaro MR Fuschi D Cremon C Carapelle M Dino P Marcellini MM. Escherichia coli Nissle 1917 restores epithelial permeability alterations induced by irritable bowel syndrome mediators. Neurogastroenterol Motil. (2018) 30:e13388. 10.1111/nmo.13388 29956419

[194] Johnson-Henry KC Donato KA Shen-Tu G Gordanpour M Sherman PM. *Lactobacillus rhamnosus* strain GG prevents enterohemorrhagic *Escherichia coli* O157:H7-induced changes in epithelial barrier function. Infect Immun. (2008) 76:1340–8. 10.1128/IAI.00778-07 18227169 PMC2292865

[195] Yu Q Yuan L Deng J Yang Q. Lactobacillus protects the integrity of intestinal epithelial barrier damaged by pathogenic bacteria. Front Cell Infect Microbiol. (2015) 5:26. 10.3389/fcimb.2015.00026 25859435 PMC4373387

[196] Zareie M Riff J Donato K McKay DM Perdue MH Soderholm JD. Novel effects of the prototype translocating *Escherichia coli*, strain C25 on intestinal epithelial structure and barrier function. Cell Microbiol. (2005) 7:1782–97. 10.1111/j.1462-5822.2005.00595.x 16309464

[197] Barbara G Feinle-Bisset C Ghoshal UC Santos J Vanner SJ Vergnolle N. The intestinal microenvironment and functional gastrointestinal disorders. Gastroenterology. (2016) 150:1305–18.e8. 10.1053/j.gastro.2016.02.028 27144620

[198] Lee M Chang EB. Inflammatory Bowel Diseases (IBD) and the microbiome—searching the crime scene for clues. Gastroenterology. (2021) 160:524–37. 10.1053/j.gastro.2020.09.056 33253681 PMC8098834

[199] Machiels K Joossens M Sabino J De Preter V Arijs I Eeckhaut V. A decrease of the butyrate-producing species *Roseburia hominis* and *Faecalibacterium prausnitzii* defines dysbiosis in patients with ulcerative colitis. Gut. (2014) 63:1275–83. 10.1136/gutjnl-2013-304833 24021287

[200] Zhou L Zhang M Wang Y Dorfman RG Liu H Yu T. *Faecalibacterium prausnitzii* produces butyrate to maintain Th17/treg balance and to ameliorate colorectal colitis by inhibiting histone deacetylase 1. Inflamm Bowel Dis. (2018) 24:1926–40. 10.1093/ibd/izy182 29796620

[201] Cremon C Guglielmetti S Gargari G Taverniti V Castellazzi AM Valsecchi C. Effect of *Lactobacillus paracasei* CNCM I-1572 on symptoms, gut microbiota, short chain fatty acids, and immune activation in patients with irritable bowel syndrome: a pilot randomized clinical trial. United Eur Gastroenterol J. (2018) 6:604–13. 10.1177/2050640617736478 29881616 PMC5987284

[202] Friedrich M Pohin M Powrie F. Cytokine networks in the pathophysiology of inflammatory Bowel disease. Immunity. (2019) 50:992–1006. 10.1016/j.immuni.2019.03.017 30995511

[203] Coccia M Harrison OJ Schiering C Asquith MJ Becher B Powrie F. IL-1β mediates chronic intestinal inflammation by promoting the accumulation of IL-17A secreting innate lymphoid cells and CD4 + Th17 cells. J Exp Med. (2012) 209:1595–609. 10.1084/jem.20111453 22891275 PMC3428945

[204] Lee YS Yang H Yang JY Kim Y Lee SH Kim JH. Interleukin-1 (IL-1) signaling in intestinal stromal cells controls KC/ CXCL1 secretion, which correlates with recruitment of IL-22- secreting neutrophils at early stages of *Citrobacter rodentium* infection. Infect Immun. (2015) 83:3257–67. 10.1128/IAI.00670-15 26034212 PMC4496604

[205] Song A Zhu L Gorantla G Berdysz O Amici SA Guerau-De-Arellano M. Salient type 1 interleukin 1 receptor expression in peripheral non-immune cells. Sci Rep. (2018) 8:723. 10.1038/s41598-018-19248-7 29335509 PMC5768710

[206] Cox CB Storm EE Kapoor VN Chavarria-Smith J Lin DL Wang L. IL-1R1-dependent signaling coordinates epithelial regeneration in response to intestinal damage. Sci Immunol. (2021) 6:eabe8856. 10.1126/sciimmunol.abe8856 33963061

[207] Madara JL Stafford J. Interferon-γ directly affects barrier function of cultured intestinal epithelial monolayers. J Clin Invest. (1989) 83:724–7. 10.1172/JCI113938 2492310 PMC303735

[208] Adams RB Planchon SM Roche JK. IFN-gamma modulation of epithelial barrier function. Time course, reversibility, and site of cytokine binding. J Immunol. (1993) 150:2356–63. 8450217

[209] Schmitz H Fromm M Bentzel CJ Scholz P Detjen K Mankertz J. Tumor necrosis factor-alpha (TNFalpha) regulates the epithelial barrier in the human intestinal cell line HT-29/B6. J Cell Sci. (1999) 112(Pt 1):137–46. 9841910 10.1242/jcs.112.1.137

[210] Bruewer M Luegering A Kucharzik T Parkos CA Madara JL Hopkins AM. Proinflammatory cytokines disrupt epithelial barrier function by apoptosis-independent mechanisms. J Immunol. (2003) 171:6164–72. 10.4049/jimmunol.171.11.6164 14634132

[211] Barbaro MR Di Sabatino A Cremon C Giuffrida P Fiorentino M Altimari A. Interferon-γ is increased in the gut of patients with irritable bowel syndrome and modulates serotonin metabolism. Am J Physiol Gastrointest Liver Physiol. (2016) 310:G439–47. 10.1152/ajpgi.00368.2015 26744473

[212] Zolotarevsky Y Hecht G Koutsouris A Gonzalez DE Quan C Tom J. A membrane-permeant peptide that inhibits MLC kinase restores barrier function in *in vitro* models of intestinal disease. Gastroenterology. (2002) 123:163–72. 10.1053/gast.2002.34235 12105845

[213] Bhat AA Uppada S Achkar IW Hashem S Yadav SK Shanmugakonar M. Tight junction proteins and signaling pathways in cancer and inflammation: a functional crosstalk. Front Physiol. (2019) 10:1942. 10.3389/fphys.2018.01942 30728783 PMC6351700

[214] Pham CTN. Neutrophil serine proteases: specific regulators of inflammation. Nat Rev Immunol. (2006) 6:541–50. 10.1038/nri1841 16799473

[215] Dale C Vergnolle N. Protease signaling to G protein-coupled receptors: implications for inflammation and pain. J Recept Signal Transduct. (2008) 28:29–37. 10.1080/10799890801941913 18437628

[216] Chin AC Lee WY Nusrat A Vergnolle N Parkos CA. Neutrophil-mediated activation of epithelial protease-activated receptors-1 and−2 regulates barrier function and transepithelial migration. J Immunol. (2008) 181:5702–10. 10.4049/jimmunol.181.8.5702 18832729 PMC2778473

[217] Barbara G Stanghellini V De Giorgio R Corinaldesi R. Functional gastrointestinal disorders and mast cells: implications for therapy. Neurogastroenterol Motil. (2006) 18:6–17. 10.1111/j.1365-2982.2005.00685.x 16371078

[218] Bashashati M Moossavi S Cremon C Barbaro MR Moraveji S Talmon G. Colonic immune cells in irritable bowel syndrome: a systematic review and meta-analysis. Neurogastroenterol Motil. (2018) 30:10. 10.1111/nmo.13192 28851005

[219] Bashashati M Rezaei N Shafieyoun A Mckernan DP Chang L Öhman L Quigley EM. Cytokine imbalance in irritable bowel syndrome: a systematic review and meta-analysis. Neurogastroenterol Motil. (2014) 26:1036–48. 10.1111/nmo.12358 24796536

[220] Chang L Adeyemo M Karagiannidis I Videlock EJ Bowe C Shih W. Serum and colonic mucosal immune markers in irritable bowel syndrome. Am J Gastroenterol. (2012) 107:262–72. 10.1038/ajg.2011.423 22158028 PMC3297737

[221] McKernan DP Gaszner G Quigley EM Cryan JF Dinan TG. Altered peripheral toll-like receptor responses in the irritable bowel syndrome. Aliment Pharmacol Ther. (2011) 33:1045–52. 10.1111/j.1365-2036.2011.04624.x 21453321

[222] Darkoh C Comer L Zewdie G Harold S Snyder N DuPont HL. Chemotactic chemokines are important in the pathogenesis of irritable bowel syndrome. PLoS ONE. (2014) 9:e93144. 10.1371/journal.pone.0093144 24667736 PMC3965506

[223] Wang F Graham WV Wang Y Witkowski ED Schwarz BT Turner JR. Interferon-γ and tumor necrosis factor-α synergize to induce intestinal epithelial barrier dysfunction by up-regulating myosin light chain kinase expression. Am J Pathol. (2005) 166:409–19. 10.1016/S0002-9440(10)62264-X 15681825 PMC1237049

[224] Hanning N Edwinson AL Ceuleers H Peters SA De Man JG Hassett LC. Intestinal barrier dysfunction in irritable bowel syndrome: a systematic review. Therap Adv Gastroenterol. (2021) 14:1756284821993586. 10.1177/1756284821993586 33717210 PMC7925957

[225] Renga G Moretti S Oikonomou V Borghi M Zelante T Paolicelli G. IL-9 and mast cells are key players of *Candida albicans* commensalism and pathogenesis in the gut. Cell Rep. (2018) 23:1767–78. 10.1016/j.celrep.2018.04.034 29742432 PMC5976578

[226] Gerlach K Hwang Y Nikolaev A Atreya R Dornhoff H Steiner S. T H 9 cells that express the transcription factor PU.1 drive T cell-mediated colitis via IL-9 receptor signaling in intestinal epithelial cells. Nat Immunol. (2014) 15:676–86. 10.1038/ni.2920 24908389

[227] Gerlach K McKenzie AN Neurath MF Weigmann B. IL-9 regulates intestinal barrier function in experimental T cell-mediated colitis. Tissue Barriers. (2015) 3:e983777. 10.4161/21688370.2014.983777 25838986 PMC4372018

[228] Piche T Barbara G Aubert P Des Varannes SB Dainese R Nano JL. Impaired Intestinal barrier integrity in the colon of patients with irritable bowel syndrome: involvement of soluble mediators. Gut. (2009) 58:196–201. 10.1136/gut.2007.140806 18824556

[229] Barbara G Stanghellini V De Giorgio R Cremon C Cottrell GS Santini D. Activated mast cells in proximity to colonic nerves correlate with abdominal pain in irritable Bowel syndrome. Gastroenterology. (2004) 126:693–702. 10.1053/j.gastro.2003.11.055 14988823

[230] Barbara G Wang B Stanghellini V de Giorgio R Cremon C Di Nardo G. Mast cell-dependent excitation of visceral-nociceptive sensory neurons in irritable Bowel syndrome. Gastroenterology. (2007) 132:26–37. 10.1053/j.gastro.2006.11.039 17241857

[231] Gecse K Róka R Ferrier L Leveque M Eutamene H Cartier C. Increased faecal serine protease activity in diarrhoeic IBS patients: a colonic lumenal factor impairing colonic permeability and sensitivity. Gut. (2008) 57:591–8. 10.1136/gut.2007.140210 18194983

[232] Pontarollo G Mann A Brandão I Malinarich F Schöpf M Reinhardt C. Protease-activated receptor signaling in intestinal permeability regulation. FEBS J. (2020) 287:645–58. 10.1111/febs.15055 31495063

[233] Barbara G Grover M Bercik P Corsetti M Ghoshal UC Ohman L. Rome foundation working team report on post-infection irritable Bowel syndrome. Gastroenterology. (2019) 156:46–58.e7. 10.1053/j.gastro.2018.07.011 30009817 PMC6309514

[234] Edogawa S Edwinson AL Peters SA Chikkamenahalli LL Sundt W Graves S Breen-Lyles M Johnson S Dyer R. Serine proteases as luminal mediators of intestinal barrier dysfunction and symptom severity in IBS. Gut. (2020) 69:62–73. 10.1136/gutjnl-2018-317416 30923071 PMC6765451

[235] Cenac N Bautzova T Le Faouder P Veldhuis NA Poole DP Rolland C. Quantification and potential functions of endogenous agonists of transient receptor potential channels in patients with irritable bowel syndrome. Gastroenterology. (2015) 149:433–4.e7. 10.1053/j.gastro.2015.04.011 25911511

[236] Bautzova T Hockley JRF Perez-Berezo T Pujo J Tranter MM Desormeaux C. 5-oxoETE triggers nociception in constipation-predominant irritable bowel syndrome through MAS-related G protein–coupled receptor D. Sci Signal. (2018) 11:eaal2171. 10.1126/scisignal.aal2171 30563864 PMC6411128

[237] Trifan A Burta O Tiuca N Petrisor DC Lenghel A Santos J. Efficacy and safety of Gelsectan for diarrhoea-predominant irritable bowel syndrome: a randomised, crossover clinical trial. United Eur Gastroenterol J. (2019) 7:1093–101. 10.1177/2050640619862721 31662866 PMC6794699

[238] Rubio-Tapia A Murray JA. Updated guidelines by the European Society for the Study of Coeliac Disease. United Eur Gastroenterol J. (2019) 7:581–2. 10.1177/2050640619849370 31210939 PMC6545712

[239] Schuppan D Junker Y Barisani D. Celiac disease: from pathogenesis to novel therapies. Gastroenterology. (2009) 137:1912–33. 10.1053/j.gastro.2009.09.008 19766641

[240] Harris LA Park JY Voltaggio L Lam-Himlin D. Celiac disease: clinical, endoscopic, and histopathologic review. Gastrointest Endosc. (2012) 76:625–40. 10.1016/j.gie.2012.04.473 22898420

[241] Greco L Romino R Coto I Di Cosmo N Percopo S Maglio M. The first large population based twin study of coeliac disease. Gut. (2002) 50:624–8. 10.1136/gut.50.5.624 11950806 PMC1773191

[242] Caminero A McCarville JL Galipeau HJ Deraison C Bernier SP Constante M. Duodenal bacterial proteolytic activity determines sensitivity to dietary antigen through protease-activated receptor-2. Nat Commun. (2019) 10:1–14. 10.1038/s41467-019-09037-9 30867416 PMC6416356

[243] Di Biase AR Marasco G Ravaioli F Dajti E Colecchia L Righi B. Gut microbiota signatures and clinical manifestations in celiac disease children at onset: a pilot study. J Gastroenterol Hepatol. (2020) 36:446–54. 10.1111/jgh.15183 32666516

[244] Marasco G Cirota GG Rossini B Lungaro L Di Biase AR Colecchia A. Probiotics, prebiotics and other dietary supplements for gut microbiota modulation in celiac disease patients. Nutrients. (2020) 12:2674. 10.3390/nu12092674 32887325 PMC7551848

[245] Stene LC Honeyman MC Hoffenberg EJ Haas JE Sokol RJ Emery L. Rotavirus infection frequency and risk of celiac disease autoimmunity in early childhood: a longitudinal study. Am J Gastroenterol. (2006) 101:2333–40. 10.1111/j.1572-0241.2006.00741.x 17032199

[246] Zafeiropoulou K Nichols B Mackinder M Biskou O Rizou E Karanikolou A. Alterations in intestinal microbiota of children with celiac disease at time of diagnosis and on a gluten-free diet. Gastroenterology. (2020) 159:2039–51.e20. 10.1053/j.gastro.2020.08.007 32791131 PMC7773982

[247] Marasco G Di Biase AR Colecchia A. Microbial signatures in celiac disease: still far from a final answer. Gastroenterology. (2020) 161:358–9. 10.1053/j.gastro.2020.10.059 33385435

[248] Marasco G Di Biase AR Schiumerini R Eusebi LH Iughetti L Ravaioli F. Gut microbiota and celiac disease. Dig Dis Sci. (2016) 61:1461–72. 10.1007/s10620-015-4020-2 26725064

[249] Jabri B Abadie V. IL-15 functions as a danger signal to regulate tissue-resident T cells and tissue destruction. Nat Rev Immunol. (2015) 15:771–83. 10.1038/nri3919 26567920 PMC5079184

[250] Catassi C Elli L Bonaz B Bouma G Carroccio A Castillejo G. Diagnosis of Non-Celiac Gluten Sensitivity (NCGS): the Salerno experts' criteria. Nutrients. (2015) 7:4966–77. 10.3390/nu7064966 26096570 PMC4488826

[251] Giovannini C Sanchez M Straface E Scazzocchio B Silano M De Vincenzi M. Induction of apoptosis in Caco-2 cells by wheat gliadin peptides. Toxicology. (2000) 145:63–71. 10.1016/S0300-483X(99)00223-1 10771132

[252] Barone MV Gimigliano A Castoria G Paolella G Maurano F Paparo F. Growth factor-like activity of gliadin, an alimentary protein: implications for coeliac disease. Gut. (2007) 56:480–8. 10.1136/gut.2005.086637 16891357 PMC1856836

[253] Heyman M Abed J Lebreton C Cerf-Bensussan N. Intestinal permeability in coeliac disease: insight into mechanisms and relevance to pathogenesis. Gut. (2012) 61:1355–64. 10.1136/gutjnl-2011-300327 21890812

[254] Clemente MG De Virgiliis S Kang JS Macatagney R Musu MP Di Pierro MR. Early effects of gliadin on enterocyte intracellular signalling involved in intestinal barrier function. Gut. (2003) 52:218–23. 10.1136/gut.52.2.218 12524403 PMC1774976

[255] Alaedini A Latov N. Transglutaminase-independent binding of gliadin to intestinal brush border membrane and GM1 ganglioside. J Neuroimmunol. (2006) 177:167–72. 10.1016/j.jneuroim.2006.04.022 16766047

[256] Bondar C Araya RE Guzman L Rua EC Chopita N Chirdo FG. Role of CXCR3/CXCL10 axis in immune cell recruitment into the small intestine in celiac disease. PLoS ONE. (2014) 9:e0089068. 10.1371/journal.pone.0089068 24586509 PMC3930692

[257] Careddu P Chiumello G Vaccari A Bardare M Zilocchi A. Effects of gluten on intestinal absorption and permeability during remission of celiac disease. Boll Soc Ital Biol Sper. (1963) 1963:1235–8. 14109593

[258] Cobden I Dickinson RJ Rothwell J Axon ATR. Intestinal permeability assessed by excretion ratios of two molecules: results in coeliac disease. Br Med J. (1978) 2:1060. 10.1136/bmj.2.6144.1060 709218 PMC1608147

[259] Oberhuber G Vogelsang H. Gastrointestinal permeability in celiac disease [1]. Gastroenterology. (1998) 114:226. 10.1016/S0016-5085(98)70661-4 9428239

[260] Van Elburg RM Uil JJ Mulder CJJ Heymans HSA. Intestinal permeability in patients with coeliac disease and relatives of patients with coeliac disease. Gut. (1993) 34:354–7. 10.1136/gut.34.3.354 8472983 PMC1374141

[261] Schulzke JD Bentzel CJ Schulzke I Riecken EO Fromm M. Epithelial tight junction structure in the jejunum of children with acute and treated celiac sprue. Pediatr Res. (1998) 43:435–41. 10.1203/00006450-199804000-00001 9544995

[262] Goswami P Das P Verma AK Prakash S Das TK Nag TC. Are alterations of tight junctions at molecular and ultrastructural level different in duodenal biopsies of patients with celiac disease and Crohn's disease? Virchows Arch. (2014) 465:521–30. 10.1007/s00428-014-1651-1 25240724

[263] Ciccocioppo R Finamore A Ara C Di Sabatino A Mengheri E Corazza GR. Altered expression, localization, and phosphorylation of epithelial junctional proteins in celiac disease. Am J Clin Pathol. (2006) 125:502–11. 10.1309/dtyr-a91g-8r0k-tm8m 16627260

[264] Montalto M Cuoco L Ricci R Maggiano N Vecchio FM Gasbarrini G. Immunohistochemical analysis of ZO-1 in the duodenal mucosa of patients with untreated and treated celiac disease. Digestion. (2002) 65:227–33. 10.1159/000063817 12239464

[265] Perry I Tselepis C Hoyland J Iqbal TH Scott D Sanders A. Reduced cadherin/catenin complex expression in celiac disease can be reproduced *in vitro* by cytokine stimulation. Lab Invest. (1999) 79:1489–99. 10616200

[266] Schumann M Siegmund B Schulzke JD Fromm M. Celiac disease: role of the epithelial barrier. CMGH. (2017) 3:150–62. 10.1016/j.jcmgh.2016.12.006 28275682 PMC5331784

[267] Mishra A Prakash S Sreenivas V Das TK Ahuja V Gupta SD. Structural and functional changes in the tight junctions of asymptomatic and serology-negative first-degree relatives of patients with celiac disease. J Clin Gastroenterol. (2016) 50:551–60. 10.1097/MCG.0000000000000436 26535478

[268] Hunt KA Zhernakova A Turner G Heap GAR Franke L Bruinenberg M. Newly identified genetic risk variants for celiac disease related to the immune response. Nat Genet. (2008) 40:395–402. 10.1038/ng.102 18311140 PMC2673512

[269] Wapenaar MC Monsuur AJ Van Bodegraven AA Weersma RK Bevova MR Linskens RK. Associations with tight junction genes PARD3 and MAGI2 in Dutch patients point to a common barrier defect for coeliac disease and ulcerative colitis. Gut. (2008) 57:463–7. 10.1136/gut.2007.133132 17989107

[270] Monsuur AJ Bakker PIWD Alizadeh BZ Zhernakova A Bevova MR Strengman E. Myosin IXB variant increases the risk of celiac disease and points toward a primary intestinal barrier defect. Nat Genet. (2005) 37:1341–4. 10.1038/ng1680 16282976

[271] Wolters VM Alizadeh BZ Weijerman ME Zhernakova A van Hoogstraten IMW Mearin ML. Intestinal barrier gene variants may not explain the increased levels of antigliadin antibodies, suggesting other mechanisms than altered permeability. Hum Immunol. (2010) 71:392–6. 10.1016/j.humimm.2010.01.016 20096742

[272] Kumar V Gutierrez-Achury J Kanduri K Almeida R Hrdlickova B Zhernakova D V. Systematic annotation of celiac disease loci refines pathological pathways and suggests a genetic explanation for increased interferon-gamma levels. Hum Mol Genet. (2015) 24:397–409. 10.1093/hmg/ddu453 25190711

[273] Almeida R Ricanõ-Ponce I Kumar V Deelen P Szperl A Trynka G. Fine mapping of the celiac disease-associated LPP locus reveals a potential functional variant. Hum Mol Genet. (2014) 23:2481–9. 10.1093/hmg/ddt619 24334606 PMC3976328

[274] Ciccocioppo R Panelli S Bellocchi MCC Cangemi GC Frulloni L Capelli E. The transcriptomic analysis of circulating immune cells in a celiac family unveils further insights into disease pathogenesis. Front Med. (2018) 5:182. 10.3389/fmed.2018.00182 29971234 PMC6018082

[275] Dolfini E Roncoroni L Elli L Fumagalli C Colombo R Ramponi S. Cytoskeleton reorganization and ultrastructural damage induced by gliadin in a three-dimensional *in vitro* model. World J Gastroenterol. (2005) 11:7597–601. 10.3748/wjg.v11.i48.7597 16437684 PMC4727224

[276] Strobel S Brydon WG Ferguson A. Cellobiose/mannitol sugar permeability test complements biopsy histopathology in clinical investigation of the jejunum. Gut. (1984) 25:1241–6. 10.1136/gut.25.11.1241 6437913 PMC1432305

[277] Gass J Bethune MT Siegel M Spencer A Khosla C. Combination enzyme therapy for gastric digestion of dietary gluten in patients with celiac sprue. Gastroenterology. (2007) 133:472–80. 10.1053/j.gastro.2007.05.028 17681168

[278] Pinier M Verdu EF Nasser-Eddine M David CS Vézina A Rivard N. Polymeric binders suppress gliadin-induced toxicity in the intestinal epithelium. Gastroenterology. (2009) 136:288–98. 10.1053/j.gastro.2008.09.016 18992747

[279] Paterson BM Lammers KM Arrieta MC Fasano A Meddings JB. The safety, tolerance, pharmacokinetic and pharmacodynamic effects of single doses of AT-1001 in coeliac disease subjects: a proof of concept study. Aliment Pharmacol Ther. (2007) 26:757–66. 10.1111/j.1365-2036.2007.03413.x 17697209

[280] Kelly CP Green PHR Murray JA Dimarino A Colatrella A Leffler DA. Larazotide acetate in patients with coeliac disease undergoing a gluten challenge: a randomised placebo-controlled study. Aliment Pharmacol Ther. (2013) 37:252–62. 10.1111/apt.12147 23163616

[281] Leffler DA Kelly CP Abdallah HZ Colatrella AM Harris LA Leon F. A randomized, double-blind study of larazotide acetate to prevent the activation of celiac disease during gluten challenge. Am J Gastroenterol. (2012) 107:1554–62. 10.1038/ajg.2012.211 22825365 PMC3463856

[282] Leffler DA Kelly CP Green PHR Fedorak RN Dimarino A Perrow W. Larazotide acetate for persistent symptoms of celiac disease despite a gluten-free diet: a randomized controlled trial. Gastroenterology. (2015) 148:1311–9.e6. 10.1053/j.gastro.2015.02.008 25683116 PMC4446229

[283] Hujoel IA Murray JA. Refractory celiac disease. Curr Gastroenterol Rep. (2020) 22:1–8. 10.1007/s11894-020-0756-8 32185560

[284] Jauregi-Miguel A. The tight junction and the epithelial barrier in coeliac disease. Int Rev Cell Mol Biol. (2021) 358:105–32. 10.1016/bs.ircmb.2020.09.010 33707052

[285] Pearson ADJ Eastham EJ Laker MF Craft AW Nelson R. Intestinal permeability in children with Crohn's disease and Coeliac disease. Br Med J. (1982) 285:20–21. 10.1136/bmj.285.6334.20 6805795 PMC1499105

[286] Ukabam SO Clamp JR Cooper BT. Abnormal small intestinal permeability to sugars in patients with Crohn's disease of the terminal ileum and colon. Digestion. (1983) 27:70–4. 10.1159/000198932 6414866

[287] Abraham C Cho JH. Mechanisms of inflammatory Bowel disease. N Engl J Med. (2009) 361:2066–78. 10.1056/NEJMra0804647 19923578 PMC3491806

[288] Miner-Williams WM Moughan PJ. Intestinal barrier dysfunction: implications for chronic inflammatory conditions of the bowel. Nutr Res Rev. (2016) 29:40–59. 10.1017/S0954422416000019 27087106

[289] Khan MW Kale AA Bere P Vajjala S Gounaris E Pakanati KC. Microbes, intestinal inflammation and probiotics. Expert Rev Gastroenterol Hepatol. (2012) 6:81–94. 10.1586/egh.11.94 22149584

[290] Ingersoll SA Ayyadurai S Charania MA Laroui H Yan Y Merlin D. The role and pathophysiological relevance of membrane transporter pept1 in intestinal inflammation and inflammatory bowel disease. Am J Physiol Gastrointest Liver Physiol. (2012) 302:G484–92. 10.1152/ajpgi.00477.2011 22194420 PMC3311434

[291] Dalmasso G Nguyen HTT Charrier-Hisamuddin L Yan Y Laroui H Demoulin B. Merlin D. PepT1 mediates transport of the proinflammatory bacterial tripeptide L-Ala-γ-D-Glu-meso-DAP in intestinal epithelial cells. Am J Physiol Gastrointest Liver Physiol. (2010) 299:687–96. 10.1152/ajpgi.00527.2009 20558765 PMC2950691

[292] Jappar D Hu Y Smith DE. Effect of dose escalation on the *in vivo* oral absorption and disposition of glycylsarcosine in wild-type and Pept1 knockout mice. Drug Metab Dispos. (2011) 39:2250–7. 10.1124/dmd.111.041087 21880829 PMC3226376

[293] De Medina FS Daddaoua A Requena P Capitán-Cañadas F Zarzuelo A Dolores Suárez M. New insights into the immunological effects of food bioactive peptides in animal models of intestinal inflammation. Proc Nutr Soc. (2010) 69:454–62. 10.1017/S0029665110001783 20598199

[294] Nässl AM Rubio-Aliaga I Sailer M Daniel H. The intestinal peptide transporter pept1 is involved in food intake regulation in mice fed a high-protein diet. PLoS ONE. (2011) 6:e0026407. 10.1371/journal.pone.0026407 22031831 PMC3198773

[295] Cipriani S Mencarelli A Chini MG Distrutti E Renga B Bifulco G. The bile acid receptor GPBAR-1 (TGR5) modulates integrity of intestinal barrier and immune response to experimental colitis. PLoS ONE. (2011) 6:e0025637. 10.1371/journal.pone.0025637 22046243 PMC3203117

[296] Chia-Hui Y. Microbiota dysbiosis and barrier dysfunction in inflammatory bowel disease: RSM Library Discovery Service. J Biomed Sci. (2018) 25:79. 10.1186/s12929-018-0483-8 30413188 PMC6234774

[297] Gruber L Lichti P Rath E Haller D. Nutrigenomics and nutrigenetics in inflammatory bowel diseases. J Clin Gastroenterol. (2012) 46:735–47. 10.1097/MCG.0b013e31825ca21a 22941427

[298] Ananthakrishnan AN Bernstein CN Iliopoulos D Macpherson A Neurath MF Ali RAR. Environmental triggers in IBD: a review of progress and evidence. Nat Rev Gastroenterol Hepatol. (2018) 15:39– 49. 10.1038/nrgastro.2017.136 29018271

[299] Ho SM Lewis JD Mayer EA Plevy SE Chuang E Rappaport SM. Challenges in IBD research: environmental triggers. Inflamm Bowel Dis. (2019) 25:S13–23. 10.1093/ibd/izz076 31095702 PMC6787673

[300] Mahid SS Minor KS Soto RE Hornung CA Galandiuk S. Smoking and inflammatory bowel disease: a meta-analysis. Mayo Clin Proc. (2006) 81:1462–71. 10.4065/81.11.1462 17120402

[301] Calkins BM. A meta-analysis of the role of smoking in inflammatory bowel disease. Dig Dis Sci. (1989) 34:1841–54. 10.1007/BF01536701 2598752

[302] Higuchi LM Khalili H Chan AT Richter JM Bousvaros A Fuchs CS. A prospective study of cigarette smoking and the risk of inflammatory bowel disease in women. Am J Gastroenterol. (2012) 107:1399–406. 10.1038/ajg.2012.196 22777340 PMC3667663

[303] Pedersen KM Çolak Y Vedel-Krogh S Kobylecki CJ Bojesen SE Nordestgaard BG. Risk of ulcerative colitis and Crohn's disease in smokers lacks causal evidence. Eur J Epidemiol. (2021) 10.1007/s10654-021-00763-3. 10.1007/s10654-021-00763-3 34091767

[304] Singh UP Singh NP Murphy EA Price RL Fayad R Nagarkatti M. Chemokine and cytokine levels in inflammatory bowel disease patients. Cytokine. (2016) 77:44–9. 10.1016/j.cyto.2015.10.008 26520877 PMC4666758

[305] Ostaff MJ Stange EF Wehkamp J. Antimicrobial peptides and gut microbiota in homeostasis and pathology. EMBO Mol Med. (2013) 5:1465–83. 10.1002/emmm.201201773 24039130 PMC3799574

[306] Salzman NH Hung K Haribhai D Chu H Karlsson-Sjöberg J Amir E. Enteric defensins are essential regulators of intestinal microbial ecology. Nat Immunol. (2010) 11:76–83. 10.1038/ni.1825 19855381 PMC2795796

[307] Peyrin-Biroulet L Beisner J Wang G Nuding S Oommen ST Kelly D. Peroxisome proliferator-activated receptor gamma activation is required for maintenance of innate antimicrobial immunity in the colon. Proc Natl Acad Sci USA. (2010) 107:8772–7. 10.1073/PNAS.0905745107 20421464 PMC2889363

[308] Wehkamp J Harder J Weichenthal M Mueller O Herrlinger KR Fellermann K. Inducible and constitutive beta-defensins are differentially expressed in Crohn's disease and ulcerative colitis. Inflamm Bowel Dis. (2003) 9:215–23. 10.1097/00054725-200307000-00001 12902844

[309] Martini E Krug SM Siegmund B Neurath MF Becker C. Mend your fences: the epithelial barrier and its relationship with mucosal immunity in inflammatory bowel disease. Cmgh. (2017) 4:33–46. 10.1016/j.jcmgh.2017.03.007 28560287 PMC5439240

[310] Courth LF Ostaff MJ Mailänder-Sánchez D Malek NP Stange EF Wehkamp J. Crohn's disease-derived monocytes fail to induce Paneth cell defensins. Proc Natl Acad Sci USA. (2015) 112:14000–5. 10.1073/pnas.1510084112 26512113 PMC4653149

[311] Wehkamp J Koslowski M Wang G Stange EF. Barrier dysfunction due to distinct defensin deficiencies in small intestinal and colonic Crohn's disease. Mucosal Immunol. (2008) 1:67–74. 10.1038/mi.2008.48 19079235

[312] Paone P Cani PD. Mucus barrier, mucins and gut microbiota: the expected slimy partners? Gut. (2020) 69:2232–43. 10.1136/gutjnl-2020-322260 32917747 PMC7677487

[313] Van Der Post S Jabbar KS Birchenough G Arike L Akhtar N Sjovall H. Structural weakening of the colonic mucus barrier is an early event in ulcerative colitis pathogenesis. Gut. (2019) 68:2142–51. 10.1136/gutjnl-2018-317571 30914450 PMC6872445

[314] Johansson ME Ambort D Pelaseyed T Schütte A Gustafsson JK Ermund A. Composition and functional role of the mucus layers in the intestine. Cell Mol Life Sci. (2011) 68:3635–41. 10.1007/S00018-011-0822-3 21947475 PMC11114784

[315] Cornick S Tawiah A Chadee K. Roles and regulation of the mucus barrier in the gut. Tissue Barriers. (2015) 3:e982426. 10.4161/21688370.2014.982426 25838985 PMC4372027

[316] Vivinus-Nébot M Frin-Mathy G Bzioueche H Dainese R Bernard G Anty R. Functional bowel symptoms in quiescent inflammatory bowel diseases: role of epithelial barrier disruption and low-grade inflammation. Gut. (2014) 63:744–52. 10.1136/gutjnl-2012-304066 23878165

[317] Hollander D Vadheim CM Brettholz E Peterson GM Delahunty T Rotter J. Increased intestinal permeability in patients with Crohn's disease and their relatives. Ann Intern Med. (1986) 105:883–5. 3777713 10.7326/0003-4819-105-6-883

[318] Arnott IDR Kingstone K Ghosh S. Abnormal intestinal permeability predicts relapse in inactive Crohn disease. Scand J Gastroenterol. (2000) 35:1163–9. 10.1080/003655200750056637 11145287

[319] Wyatt J Vogelsang H Hübl W Waldhoer T Lochs H. Intestinal permeability and the prediction of relapse in Crohn's disease. Lancet. (1993) 341:1437–9. 10.1016/0140-6736(93)90882-H 8099141

[320] Arrieta MC Bistritz L Meddings JB. Alterations in intestinal permeability. Gut. (2006) 55:1512–20. 10.1136/gut.2005.085373 16966705 PMC1856434

[321] Madsen KL Malfair D Gray D Doyle JS Jewell LD Fedorak RN. Interleukin-10 gene-deficient mice develop a primary intestinal permeability defect in response to enteric microflora. Inflamm Bowel Dis. (1999) 5:262–70. 10.1097/00054725-199911000-00004 10579119

[322] Reuter BK Pizarro TT. Mechanisms of tight junction dysregulation in the SAMP1YitFc model of Crohn's disease-like ileitis. Ann N Y Acad Sci. (2009) 1165:301–7. 10.1111/j.1749-6632.2009.04035.x 19538320

[323] Su L Shen L Clayburgh DR Nalle SC Sullivan EA Jon B. Activation and contributes to development of experimental colitis. Gastroenterology. (2010) 136:551–63. 10.1053/j.gastro.2008.10.081 19027740 PMC2712351

[324] Blair SA Kane S V. Clayburgh DR Turner JR. Epithelial myosin light chain kinase expression and activity are upregulated in inflammatory bowel disease. Lab Investig. (2006) 86:191–201. 10.1038/labinvest.3700373 16402035

[325] Swidsinski A Ladhoff A Pernthaler A Swidsinski S Loening Baucke V Ortner M. Mucosal flora in inflammatory bowel disease. Gastroenterology. (2002) 122:44–54. 10.1053/gast.2002.30294 11781279

[326] Prasad S Mingrino R Kaukinen K Hayes KL Powell RM MacDonald TT. Inflammatory processes have differential effects on claudins 2, 3 and 4 in colonic epithelial cells. Lab Investig. (2005) 85:1139–62. 10.1038/labinvest.3700316 16007110

[327] Ménard S Cerf-Bensussan N Heyman M. Multiple facets of intestinal permeability and epithelial handling of dietary antigens. Mucosal Immunol. (2010) 3:247–59. 10.1038/mi.2010.5 20404811

[328] Oshima T Laroux FS Coe LL Morise Z Kawachi S Bauer P. Interferon-γ and interleukin-10 reciprocally regulate endothelial junction integrity and barrier function. Microvasc Res. (2001) 61:130–43. 10.1006/mvre.2000.2288 11162203

[329] Albert-Bayo M Paracuellos I González-Castro AM Rodríguez-Urrutia A Rodríguez-Lagunas MJ Alonso-Cotoner C. Intestinal mucosal mast cells: key modulators of barrier function and homeostasis. Cells. (2019) 8:135. 10.3390/cells8020135 30744042 PMC6407111

[330] Al-Sadi R Ye D Boivin M Guo S Hashimi M Ereifej L. Interleukin-6 modulation of intestinal epithelial tight junction permeability is mediated by JNK pathway. PLoS ONE. (2014) 9:e0085345. 10.1371/journal.pone.0085345 24662742 PMC3963839

[331] Gassler N Rohr C Schneider A Kartenbeck J Bach A Obermüller N. Inflammatory bowel disease is associated with changes of enterocytic junctions. Am J Physiol Gastrointest Liver Physiol. (2001) 281:216–28. 10.1152/ajpgi.2001.281.1.g216 11408275

[332] Shih DQ Michelsen KS Barrett RJ Biener-Ramanujan E Gonsky R Zhang X. Insights into TL1A and IBD pathogenesis. Adv Exp Med Biol. (2011) 691:279–88. 10.1007/978-1-4419-6612-4_29 21153332

[333] Cooney R Jewell D. The genetic basis of inflammatory bowel disease. Dig Dis. (2009) 27:428–42. 10.1159/000234909 19897957

[334] Ishihara S Aziz MM Yuki T Kazumori H Kinoshita Y. Inflammatory bowel disease: review from the aspect of genetics. J Gastroenterol. (2009) 44:1097–108. 10.1007/s00535-009-0141-8 19802731

[335] Mayer L. Evolving paradigms in the pathogenesis of IBD. J Gastroenterol. (2010) 45:9–16. 10.1007/s00535-009-0138-3 19960355

[336] Hugot JP Chamaillard M Zouali H Lesage S Cézard JP Belaiche J. Association of NOD2 leucine-rich repeat variants with susceptibility to Crohn's disease. Nature. (2001) 411:599–603. 10.1038/35079107 11385576

[337] Kosovac K Brenmoehl J Holler E Falk W Schoelmerich J Hausmann M. Association of the NOD2 genotype with bacterial translocation via altered cell-cell contacts in Crohn's disease patients. Inflamm Bowel Dis. (2010) 16:1311–21. 10.1002/ibd.21223 20232407

[338] Rosenstiel P Sebreiber S. NOD-like receptors-pivotal guardians of the immunological integrity of barrier organs. Adv Exp Med Biol. (2009) 653:35–47. 10.1007/978-1-4419-0901-5_3 19799110

[339] Girardin SE Boneca IG Viala J Chamaillard M Labigne A Thomas G. Nod2 is a general sensor of peptidoglycan through muramyl dipeptide (MDP) detection. J Biol Chem. (2003) 278:8869–72. 10.1074/jbc.C200651200 12527755

[340] Inohara N Ogura Y Fontalba A Gutierrez O Pons F Crespo J. Host recognition of bacterial muramyl dipeptide mediated through NOD2: implications for Crohn's disease. J Biol Chem. (2003) 278:5509–12. 10.1074/jbc.C200673200 12514169

[341] Rosenstiel P Fantini M Bräutigam K Kühbacher T Waetzig GH Seegert D. TNF-α and IFN-γ regulate the expression of the NOD2 (CARD15) gene in human intestinal epithelial cells. Gastroenterology. (2003) 124:1001–9. 10.1053/gast.2003.50157 12671897

[342] Buhner S Buning C Genschel J Kling K Herrmann D Dignass A. Genetic basis for increased intestinal permeability in families with Crohn's disease: role of CARD15 3020insC mutation? Gut. (2006) 55:342–7. 10.1136/gut.2005.065557 16000642 PMC1856071

[343] Matsuoka K Kanai T. The gut microbiota and inflammatory bowel disease. Semin Immunopathol. (2015) 37:47–55. 10.1007/s00281-014-0454-4 25420450 PMC4281375

[344] Fukata M Arditi M. The role of pattern recognition receptors in intestinal inflammation. Mucosal Immunol. (2013) 6:451–63. 10.1038/mi.2013.13 23515136 PMC3730813

[345] Zeuthen LH Fink LN Frokiaer H. Epithelial cells prime the immune response to an array of gut-derived commensals towards a tolerogenic phenotype through distinct actions of thymic stromal lymphopoietin and transforming growth factor-β. Immunology. (2008) 123:197–208. 10.1111/j.1365-2567.2007.02687.x 17655740 PMC2433297

[346] Rimoldi M Chieppa M Salucci V Avogadri F Sonzogni A Sampietro GM. Intestinal immune homeostasis is regulated by the crosstalk between epithelial cells and dendritic cells. Nat Immunol. (2005) 6:507–14. 10.1038/ni1192 15821737

[347] Travis S Menzies I. Intestinal permeability: functional assessment and significance. Clin Sci. (1992) 82:471–88. 10.1042/cs0820471 1317756

[348] Bjarnason I Macpherson A Hollander D. Intestinal permeability: an overview. Gastroenterology. (1995) 108:1566–81. 10.1016/0016-5085(95)90708-4 7729650

[349] Wehkamp J Stange EF. Paneth's disease. J Crohn's Colitis. (2010) 4:523–31. 10.1016/j.crohns.2010.05.010 21122555

[350] Khoshbin K Camilleri M. Effects of dietary components on intestinal permeability in health and disease. Am J Physiol Gastrointest Liver Physiol. (2020) 319:G589–608. 10.1152/AJPGI.00245.2020 32902315 PMC8087346

[351] Camilleri M. Human intestinal barrier: effects of stressors, diet, prebiotics, and probiotics. Clin Transl Gastroenterol. (2021) 12:e00308. 10.14309/ctg.0000000000000308 33492118 PMC7838004

[352] Klimberg VS Souba WW. The importance of intestinal glutamine metabolism in maintaining a healthy gastrointestinal tract and supporting the body's response to injury and illness. Surg Annu. (1990) 22:61–76. 2408172

[353] Zhou YP Jiang ZM Sun YH Wang XR Ma EL Wilmore D. The effect of supplemental enteral glutamine on plasma levels, gut function, and outcome in severe burns: a randomized, double-blind, controlled clinical trial. J Parenter Enter Nutr. (2003) 27:241–5. 10.1177/0148607103027004241 12903886

[354] Peng X Yan H You Z Wang P Wang S. Effects of enteral supplementation with glutamine granules on intestinal mucosal barrier function in severe burned patients. Burns. (2004) 30:135–9. 10.1016/j.burns.2003.09.032 15019120

[355] Zhou QQ Verne ML Fields JZ Lefante JJ Basra S Salameh H. Randomised placebo-controlled trial of dietary glutamine supplements for postinfectious irritable bowel syndrome. Gut. (2019) 68:996–1002. 10.1136/gutjnl-2017-315136 30108163 PMC9549483

[356] Norman AW. From vitamin D to hormone D: fundamentals of the vitamin D endocrine system essential for good health. Am J Clin Nutr. (2008) 88:491–9S. 10.1093/ajcn/88.2.491s 18689389

[357] Kong J Zhang Z Musch MW Ning G Sun J Hart J. Novel role of the vitamin D receptor in maintaining the integrity of the intestinal mucosal barrier. Am J Physiol Gastrointest Liver Physiol. (2007) 294:G208–16. 10.1152/ajpgi.00398.2007 17962355

[358] Hewison M. Vitamin D and innate and adaptive immunity. Vitam Horm. (2011) 86:23–62. 10.1016/B978-0-12-386960-9.00002-2 21419266

[359] Froicu M Cantorna MT. Vitamin D and the vitamin D receptor are critical for control of the innate immune response to colonic injury. BMC Immunol. (2007) 8:5. 10.1186/1471-2172-8-5 17397543 PMC1852118

[360] Zhao H Zhang H Wu H Li H Liu L Guo J. Protective role of 1,25(OH)2vitamin D3 in the mucosal injury and epithelial barrier disruption in DSS-induced acute colitis in mice. BMC Gastroenterol. (2012) 12:57. 10.1186/1471-230X-12-57 22647055 PMC3464614

[361] Guzman-Prado Y Samson O Segal JP Limdi JK Hayee B. Vitamin D therapy in adults with inflammatory bowel disease: a systematic review and meta-analysis. Inflamm Bowel Dis. (2020) 26:1819–30. 10.1093/ibd/izaa087 32385487

[362] Raftery T Martineau AR Greiller CL Ghosh S McNamara D Bennett K. Effects of vitamin D supplementation on intestinal permeability, cathelicidin and disease markers in Crohn's disease: results from a randomised double-blind placebo-controlled study. United Eur Gastroenterol J. (2015) 3:294–302. 10.1177/2050640615572176 26137304 PMC4480538

[363] Corrêa-Oliveira R Fachi JL Vieira A Sato FT Vinolo MAR. Regulation of immune cell function by short-chain fatty acids. Clin Transl Immunol. (2016) 5:e73. 10.1038/cti.2016.17 27195116 PMC4855267

[364] Ríos-Covián D Ruas-Madiedo P Margolles A Gueimonde M De los Reyes-Gavilán CG Salazar N. Intestinal short chain fatty acids and their link with diet and human health. Front Microbiol. (2016) 7:185. 10.3389/fmicb.2016.00185 26925050 PMC4756104

[365] Barcenilla A Pryde SE Martin JC Duncan SH Stewart CS Henderson C. Phylogenetic relationships of butyrate-producing bacteria from the human gut. Appl Environ Microbiol. (2000) 66:1654–61. 10.1128/AEM.66.4.1654-1661.2000 10742256 PMC92037

[366] Louis P Young P Holtrop G Flint HJ. Diversity of human colonic butyrate-producing bacteria revealed by analysis of the butyryl-CoA:acetate CoA-transferase gene. Environ Microbiol. (2010) 12:304–14. 10.1111/j.1462-2920.2009.02066.x 19807780

[367] Vital M Howe AC Tiedje JM. Revealing the bacterial butyrate synthesis pathways by analyzing (meta)genomic data. MBio. (2014) 5:e00889. 10.1128/mBio.00889-14 24757212 PMC3994512

[368] Kannampalli P Shaker R Sengupta JN. Colonic butyrate- algesic or analgesic? Neurogastroenterol Motil. (2011) 23:975–9. 10.1111/j.1365-2982.2011.01775.x 21981302 PMC3191935

[369] Banasiewicz T Krokowicz Stojcev Z Kaczmarek BF Kaczmarek E Maik J. Microencapsulated sodium butyrate reduces the frequency of abdominal pain in patients with irritable bowel syndrome. Color Dis. (2013) 15:204–9. 10.1111/j.1463-1318.2012.03152.x 22738315

