# Corrigendum: Inflammatory and Microbiota-Related Regulation of the Intestinal Epithelial Barrier

**DOI:** 10.3389/fnut.2021.790387

**Published:** 2021-11-01

**Authors:** Giovanni Barbara, Maria Raffaella Barbaro, Daniele Fuschi, Marta Palombo, Francesca Falangone, Cesare Cremon, Giovanni Marasco, Vincenzo Stanghellini

**Affiliations:** ^1^IRCCS Azienda Ospedaliero-Universitaria di Bologna, Bologna, Italy; ^2^Department of Medical and Surgical Sciences, University of Bologna, Bologna, Italy; ^3^Medical-Surgical Department of Clinical Sciences and Translational Medicine, University Sapienza, Rome, Italy

**Keywords:** intestinal epithelial barrier, mucosal immune system, gut microbiota, IBS, IBD, celiac disease, non-celiac gluten sensitivity

**Incorrect Reference**

In the original article, there is a mistake in the references cited in the text. From reference 105 onwards, the number does not correspond to the correct citation. The corrected references appear below.

The authors apologize for this error and state that this does not change the scientific conclusions of the article in any way. The original article has been updated.

## Publisher's Note

All claims expressed in this article are solely those of the authors and do not necessarily represent those of their affiliated organizations, or those of the publisher, the editors and the reviewers. Any product that may be evaluated in this article, or claim that may be made by its manufacturer, is not guaranteed or endorsed by the publisher.
